# Two telomere‐to‐telomere pig genome assemblies and pan‐genome analyses provide insights into genomic structural landscape and genetic adaptations

**DOI:** 10.1002/imt2.70013

**Published:** 2025-04-03

**Authors:** Wencheng Zong, Li Chen, Dongjie Zhang, Yuebo Zhang, Jinbu Wang, Xinhua Hou, Jie Chai, Yalong An, Ming Tian, Xinmiao He, Chengyi Song, Jun He, Xin Liu, Ligang Wang, Enrico D'Alessandro, Lixian Wang, Yulong Yin, Mingzhou Li, Di Liu, Jinyong Wang, Longchao Zhang

**Affiliations:** ^1^ State Key Laboratory of Animal Biotech Breeding, Institute of Animal Sciences Chinese Academy of Agricultural Sciences (CAAS) Beijing China; ^2^ National Center of Technology Innovation for Pigs Chongqing Academy of Animal Science Chongqing China; ^3^ Institute of Animal Husbandry Heilongjiang Academy of Agricultural Sciences Harbin China; ^4^ Yuelushan laboratory Changsha China; ^5^ Key Laboratory of Livestock and Poultry Resources (Pig) Evaluation and Utilization, Ministry of Agriculture and Rural Affairs, College of Animal Science and Technology Hunan Agricultural University Changsha China; ^6^ Key Laboratory of Animal Genetics, Breeding and Reproduction of Shaanxi Province, College of Animal Science and Technology Northwest A&F University Xianyang China; ^7^ College of Animal Science and Technology Yangzhou University Yangzhou China; ^8^ Department of Veterinary Science, Division of Animal Production University of Messina Messina Italy; ^9^ State Key Laboratory of Swine and Poultry Breeding Industry, College of Animal Science and Technology Sichuan Agricultural University Chengdu China

## Abstract

This study presented two high‐precision telomere‐to‐telomere genome assemblies for Min and Rongchang pigs, including a detailed exploration of the telomeric and centromeric regions. By integrating pan‐genome and multi‐omics analyses, structural variations linked to genetic adaptation were identified, providing a valuable resource for advancing pig breeding and genetic improvement.

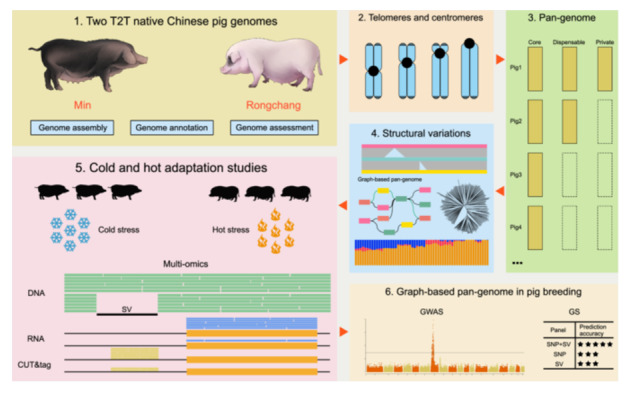

To the Editor,

*Sus scrofa* (i.e., pig or swine) is of enormous agricultural significance and serves as an attractive biomedical model [1]. China's ecological diversity has given rise to native pig breeds with remarkable phenotypic and genetic traits [2, 3]. Two geographically distant native Chinese breeds—the Min pig from the Northeast and the Rongchang pig from the Southwest—have adapted to cold and hot environments, respectively, through distinct selective pressures.

The initial pig genome map was constructed using a bacterial artificial chromosome clone library for the Duroc breed [4]. Next‐generation sequencing data led to the release of the Sscrofa10.2 genome [5]. Third‐generation sequencing further facilitated the release of the more contiguous Sscrofa11.1 genome [6], which serves as the cornerstone of current pig genomics research. Despite several existing genome assemblies [7, 8, 9, 10], a telomere‐to‐telomere (T2T) genome for pigs was still lacking.

This study presents the T2T genome assemblies for Min and Rongchang pigs (T2T_Mpig1.0 and T2T_RCpig1.0), offering the most advanced pig genome versions to date. It allows for the detailed analysis of telomeric and centromeric regions. A pan‐genome analysis of gene families and structural variations (SVs) across breeds was performed, integrating multi‐omics data to assess the role of SVs in genetic adaptation of pigs to environmental stresses. These findings highlight the importance of SVs in pig breeding and provide key genomic resources for future pig genetic improvements.

## RESULTS AND DISCUSSION

### T2T genome assemblies of two native Chinese pig breeds

**Figure 1:**
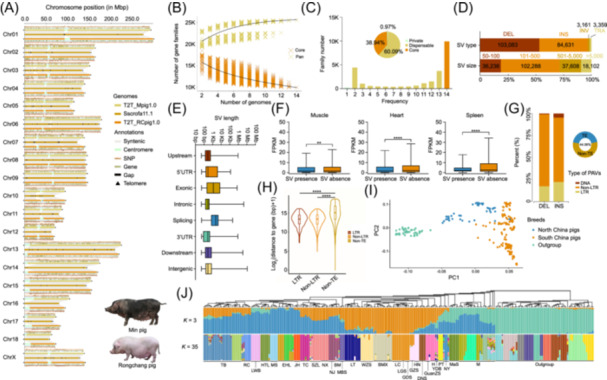
Two T2T pig genome assemblies and pan‐genome studies. (A) A synteny plot of the comparison between two T2T genomes with Sscrofa11.1 (the reference pig genome). T2T_Mpig1.0 and T2T_RCpig1.0 denote the T2T genomes of Min and Rongchang pigs, respectively. (B) The dynamics of gene families in the pan‐ and core genomes are shown with the addition of new pig genomes. (C) Pan‐genome composition and frequency. The histogram illustrates the number of gene families across 14 genomes with varying frequencies. The pie chart indicates the proportion of gene families corresponding to each category. (D) The percentage of SV types and sizes. (E) The distribution of SV lengths across different genomic regions. (F) A comparison of gene expression between the presence and absence of SVs. The edges and centerlines of the boxes represent the interquartile range (IQR) and medians, while the whiskers extend to 1.5 × IQR. (G) The type and percentage of TE‐PAVs. The doughnut chart displays the proportion of PAVs originating from TEs. (H) The distance of PAVs from their closest protein‐coding genes. Analyses of (I) two‐way PCA, (J) phylogenetic tree, and the genetic structure of 246 pigs collected from 35 breeds. The Western pigs were used as the outgroup. For (F) and (H), a two‐sided Wilcoxon test was used to assess statistical significance. Statistical significance was indicated as follows: *p < 0.05, **p < 0.01, ***p < 0.001, ****p < 0.0001. BM, Bamei; BMX, Bama Xiang; DNS, Diannan small‐ear; EHL, Erhualian; GDS, Guangdong small‐ear; GZS, Guizhong spotted; GuanZS, Guanzhuang spotted; HN, Hainan; H, Huai; HTL, Hetao large‐ear; JH, Jinhua; LC, Luchuan; LGS, Liangguang small‐ear spotted; LT, Lantang; LWB, Laiwu black; MaS, Mashen; MBS, Minbei spotted; M, Min; MS, Meishan; NJ, Neijiang; NX, Ningxiang; NY, Nanyang; PT, Putian; RC, Rongchang; SZL, Shaziling; TB, Tibetan; TC, Tongcheng; WZS, Wuzhishan; YDB, Yuedong black.

By integrating multiple sequencing platforms (Table S1) and assembly algorithms, we generated the high‐quality T2T genome assemblies for the Min pig (T2T_Mpig1.0, completely gapless, with a contig N50 size of ~143.37 Mb, and a total genome size of ~2.66 Gb) and the Rongchang pig (T2T_RCpig1.0, containing a single gap located within the repetitive regions, with a contig N50 size of ~143.96 Mb, and a total genome size of ~2.68 Gb) (Figures 1A, and S1, Tables S2 and S3). The chromatin interaction maps showed strong intra‐chromosomal interactions, confirming the accuracy of genome assembly (Figure S2). We identified 182.94 Mb of previously unresolved regions, which are primarily composed of repeats (78.82%), with 75.56% being tandem repeats (TRs) and 24.44% being dispersed repeats. This is most likely attributed to the incomplete assembly of telomeric and centromeric regions (Figure 1A).

Repetitive sequences constituted 43.52% (~1.16 Gb) of the T2T_Mpig1.0 and 44.09% (~1.18 Gb) of the T2T_RCpig1.0 genomes (Table S4). After repeat masking, the genomes were annotated using homology‐based, transcriptome‐based, and *ab initio* approaches. A total of 25,054 and 26,816 protein‐coding genes were predicted in T2T_Mpig1.0 and T2T_RCpig1.0, respectively (Table S5). Fourteen novel genes that were absent in Sscrofa11.1 were found in both genomes (Table S6). Noncoding RNA annotations included 7636 and 7569 miRNAs, 4531 and 4542 tRNAs, 769 and 617 rRNAs, and 2251 and 2384 snRNAs in T2T_Mpig1.0 and T2T_RCpig1.0, respectively.

### Quality assessment of two pig genome assemblies

Building on the high‐quality genome assemblies, the quality of the genomes was further assessed. We aligned Illumina and HiFi reads to their respective genomes and obtained high mapping ratios (over 99.73%) and coverage ratios (over 99.95%). BUSCO analysis showed completeness scores of ~98.2% for T2T_Mpig1.0 and ~98.3% for T2T_RCpig1.0, respectively (Figure S3). Base accuracy, assessed via homozygous single nucleotide variants (SNVs), revealed low error ratios (3.39 × 10^−^^6^ for T2T_Mpig1.0, 4.78 × 10^−^^6^ for T2T_RCpig1.0). Merqury analysis of the Illumina reads yielded quality values (QV) of 51.17 and 47.84, with error ratios of 7.64 × 10^−^^6^ and 1.65 × 10^−^^5^, reflecting an accuracy of over 99.998% (Table S7). The annotation of protein‐coding genes showed 93.96% and 91.15% of the genes in T2T_Mpig1.0 and T2T_RCpig1.0, respectively, were annotated as orthologs, confirming a high annotation precision (Table S8).

### Telomeres and centromeres of the two T2T pig genomes

Telomeres were identified at the termini of each of the 19 chromosomes (Figure S4, Table S9). By searching for candidate TRs, we identified potential centromeric regions, with 987 and 1117 unique repeats found in T2T_Mpig1.0 and T2T_RCpig1.0 genomes, respectively. The most abundant repeats were 336 bp in length for the two genomes (Figure S5). Centromeres were located based on the positions of genes and transposable elements (TEs), with chromosomes 1–12 and X being metacentric/submetacentric, and chromosomes 13–18 being acrocentric/telocentric (Table S10). The composition of TRs varied with centromere position, showing relative conservation between the genomes, with specific repeat patterns identified on different chromosomes (Figures S6 and S7).

### Construction of the gene‐based pan‐genome in pigs

We performed a pan‐genome analysis of protein‐coding genes by integrating our two T2T gene annotations with 12 publicly available gene annotations (Table S11), resulting in 277,780 genes being clustered into 25,472 nonredundant gene families (Table S12). The incorporation of additional genomes underscored the significant genetic diversity existing within the pig population (Figures 1B and S8). Core gene families accounted for 38.94% of the total gene families, dispensable ones 60.09%, and private ones 0.97% (Figure 1C). On average, each genome contained 54.68% core, 45.22% dispensable, and 1.41% private gene families (Figure S9, Table S13). The T2T genomes unveiled an increase in the number of gene families, with a particularly significant increase in dispensable ones.

Core genes showed higher expression, longer coding sequences, and lower Ka/Ks ratios and nucleotide diversity (π) (Figure S10A–D), indicating stable selection. In contrast, dispensable and private genes were subjected to more intense evolutionary pressures. GO and KEGG analyses showed that core genes are involved in essential processes such as transcriptional regulation and immune responses (Figure S11A,D). Dispensable genes are associated with cellular functions, metabolism and olfactory transduction (Figure S11B,E), while private genes are enriched in pathways related to UV protection, fatty acid β‐oxidation, and specific metabolic processes (Figure S11C,F).

### SVs in pig genomes

The T2T genomes provide a comprehensive framework for detecting genome‐wide SVs. By integrating the genomes and re‐sequencing data of multiple pig breeds from North America, Europe, and East Asia (Figure S12, Tables S14 and S15), a total of 194,234 SVs were identified, with 81.79% (158,858) derived from genome alignment and 18.21% (35,376) from re‐sequencing data (Figure S13). These SVs included 103,083 deletions (DELs), 84,631 insertions (INSs), 3161 inversions (INVs), and 3359 translocations (TRAs) (Figures 1D), and 96.64% of the variations were presence/absence variations (PAVs). Most variants (90.68%) were smaller than 5000 bp, with the highest frequency in the 101–500 bp range (52.66%) (Figure 1D). SVs were primarily located in intronic and intergenic regions, with only 2.53%, 0.21%, 0.46%, and 0.18% located within 2 kb upstream or downstream of genes, untranslated regions (UTRs), exons, and splice regions, respectively. Notably, SVs in the 5' UTR, exonic, and splice regions were generally larger in length (Figure 1E). Supporting the notion that SVs impact gene expression through dosage effects, genes in regions with SVs (upstream and downstream genes within 2 kb, introns, exons, splice sites, and UTRs) were significantly downregulated in multiple tissues compared to those without SVs (Figure 1F). A significant portion of PAVs (44.06%) originated from TEs (TE‐PAVs), with non‐LTR TEs accounting for 81.87% of DELs and 73.36% of INSs, and LTR TEs accounting for 17.22% of DELs and 21.99% of INSs (Figure 1G). TE‐PAVs were located closer to coding genes than non‐TE‐PAVs (Figure 1H).

A graph pan‐genome was constructed using T2T_Mpig1.0 and all PAVs, and 246 high‐depth sequencing samples (~20× depth) (Table S16) were genotyped. Principal component analysis confirmed that native Chinese pig breeds were genetically distinct from Western commercial pig breeds, and there was further geographic differentiation within Chinese pig breeds (Figure 1I). Phylogenetic and population structure analyses further confirmed the accuracy of genotyping, consistently clustering geographically neighboring breeds together (Figure 1J).

### Functionally relevant variations under temperature stress conditions

**Figure 2:**
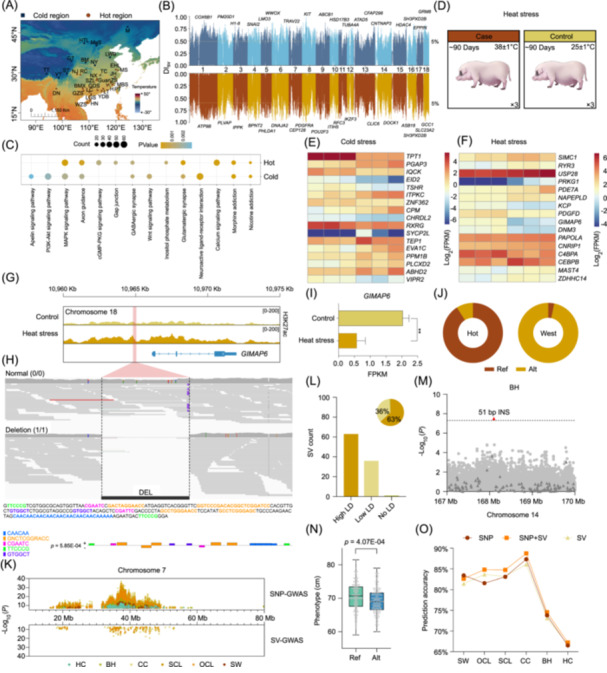
Multi‐omics analyses of temperature adaptation and a graph‐based pan‐genome approach in pig breeding. (A) The geographical distribution of pig breeds in cold and hot regions. (B) Manhattan plots of the DI_SV_ statistics for the separated comparison of pig breeds residing in the cold region (*upper* panel) and the hot region (*lower* panel) with Western commercial pig breeds, based on the SVs. The dashed line indicates the DI_SV_ statistic for the Top 5%, and genes associated with the most significant SVs on each chromosome are labeled. (C) KEGG analysis of genes associated with loci within the Top 5% of DI_SV_ statistics. Only pathways with *p* < 0.05 were retained. (D) A schematic diagram of the Rongchang pig heat stress experiment. Differentially expressed genes overlapping with the Top 5% population‐stratified SVs under cold stress in (E) Min pigs and heat stress in (F) Rongchang pigs. (G) Genome browser visualization of CUT&Tag data for the *GIMAP6* locus in skeletal muscle of Rongchang pig. The position marked by a red rectangle designates the location of the DEL. (H) Visualization of the position, sequence, and motif for the DEL upstream of *GIMAP6*. (I) Comparison of the expression of *GIMAP6* between heat stress and control conditions. A Student's *t*‐test was used to assess statistical significance. Statistical significance was indicated as follows: *p < 0.05, **p < 0.01, ***p < 0.001, ****p < 0.0001. (J) Genotype frequencies at the DEL upstream of the *GIMAP6* were compared between pigs from hot regions and Western commercial breeds. (K) Significant SV and SNP signals were detected on chromosome 7. Phenotypic abbreviations: SW (slaughter weight), OCL (oblique carcass length), SCL (straight carcass length), CC (cannon circumference), BH (body height), and HC (hip circumference). (L) Linkage disequilibrium between SV and surrounding SNPs within a 500‐kb range. (M) A localized Manhattan plot for body height. Triangles represent SVs, while points denote SNPs. (N) The box plot displays the phenotypic differences between the reference and alternative alleles. (O) GS for six phenotypes with different markers.

To investigate the SVs associated with the unique adaptations of native Chinese pigs, we compared 72 individuals from cold regions and 112 from hot regions with Western commercial breeds (Figure 2A). Population‐stratified SVs were identified using the DI_SV_ method, focusing on the top 5% of SVs with the highest DI_SV_ values (Figure 2B). Among these, 4241 SVs overlapped with 2271 functional genes in the cold group and 2331 in the heat group, suggesting selective pressure during domestication. These genes are involved in signaling pathways (e.g., apelin, PI3K‐Akt, and MAPK) and neurological pathways (e.g., GABAergic, glutamatergic synapses, and nicotine addiction) (Figure 2C, Table S17).

To explore gene expression changes under extreme temperature conditions, the RNA‐seq data derived from a heat stress experiment on the hot‐adapted group (Rongchang pigs) (Figures 2D and S14, Table S18) were compared with a previously reported cold stress experiment on the cold‐adapted group (Min pigs) [11]. In the cold‐adapted group, 204 differentially expressed genes (DEGs) were identified (Log2FC > |0.59|, *p*adj < 0.05), with 116 genes downregulated and 88 genes upregulated (Figure S15A, Table S19). In contrast to an earlier study [11], our advanced T2T genome revealed 89 genes that had not been previously characterized in the context of cold stress (Figure S16). Notably, the *TPT1* gene, which is associated with adipogenic differentiation [12], was significantly downregulated (*p*adj = 4.37E‐34). In the heat‐adapted group, 259 DEGs were identified, with 146 genes upregulated and 113 genes downregulated (Figure S15B, Table S20). Multiple heat shock proteins and solute carriers were implicated in the stress response. GO and KEGG pathway annotations showed that the cold‐adapted DEGs were involved in tissue remodeling, immune regulation, and cellular stress responses (Figure S17A,B), while hot‐adapted DEGs were related to protein folding, immune response, and glucose metabolism (Figure S17C,D).

The SV and RNA‐seq data were integrated to identify candidate SVs that potentially contribute to temperature responses. Candidate SVs were defined as those in the top 5% of DI_SV_ values and overlapping with DEGs within a 5 kb range upstream or downstream. In the cold‐adapted group, 17 SV‐associated genes were identified (Figure 2E), while in the heat‐adapted group, 16 such genes were identified (Figure 2F).

To investigate the regulatory role of these loci, CUT&Tag sequencing with antibodies against H3K27ac was performed on heat‐adapted group (Figures S18 and S19). A total of 6271 differential peaks (Log2FC > |0.59|, *p* < 0.05) were identified (Table S21). Among the 16 candidate SVs, a 226 bp DEL overlapping a differential peak ~1 kb upstream of *GIMAP6* was identified (Figure 2G). This DEL was confirmed through re‐sequencing data and gel electrophoresis (Figures 2H and S20). *GIMAP6*, which is involved in immune function and dysregulation [13], was downregulated after heat exposure (*t*‐test, *p* = 1.71E‐03) (Figure 2I). A previous study showed that the downregulation of *GIMAP6* could increase apoptotic sensitivity and accelerate T‐cell activation [14]. The genotype frequencies differed significantly between hot region population (91.07%) and commercial breeds (3.23%) (Figure 2J). The DEL sequence contained five motifs: CAACAA, GNCTCGGRACC, CGAATC, TTCCCG, and GTGGCT (Figure 2H). GO analysis revealed that the associated genes are involved in sensory perception, immune response regulation, and development (Figure S21). These findings suggest that this DEL may regulate the expression of *GIMAP6* and contribute to adaptation to extreme temperatures.

### A graph‐based pan‐genome approach in pig breeding

Graph pan‐genomes have improved QTL identification and genomic selection (GS) in crops [15, 16], but their application in animal breeding is limited. To evaluate the potential of SVs in pig breeding, re‐sequencing data from 540 individuals in the Large White × Min pig *F*_*2*_ population [17] were genotyped using an established graph pan‐genome. Genome‐wide association studies (GWAS) and GS for six carcass traits revealed significant associations on chromosome 7 (Figures 2K and S22), with SNPs and SVs contributing 40,451 and 198 signals, respectively. On average, 503 SNPs were located within 500‐kb of each SV, with 63% showing strong linkage (*R*² > 0.6) (Figure 2L). A 56‐bp INS on chromosome 14 (*p* = 3.79E‐08) showed significant phenotypic differences (*t*‐test, *p* = 4.07E‐04) but had no association with SNPs (Figure 2M,N), indicating that some genetic information is only captured through SVs. The predicted GS accuracies varied by phenotypes, with SNP panels outperforming for SW, CC, and BH, while SV panels were better for OCL, SCL, and HC (Figure 2O). The combination of SNP + SV panels improved the accuracy by 1%–4% for over 80% of phenotypes. Our analyses showed that incorporating SVs into breeding studies enhanced the resolution of GWAS and the accuracy of GS, offering valuable insights for the development of molecular markers in pig breeding.

## METHODS

Detailed procedures for biological sample collection, the sequencing protocol, data processing techniques for sequencing data, and bioinformatic and statistical analysis approaches are available in the Supplementary Information.

## AUTHOR CONTRIBUTIONS

**Wencheng Zong**: Conceptualization; methodology; software; data curation; formal analysis; investigation; writing—original draft; writing—review and editing; visualization; validation. **Li Chen**: Conceptualization; methodology; software; formal analysis; resources. **Dongjie Zhang**: Conceptualization; methodology; software; formal analysis; resources. **Yuebo Zhang**: Conceptualization; methodology; software; formal analysis. **Jinbu Wang**: Methodology; software; visualization. **Xinhua Hou**: Methodology; investigation. **Jie Chai**: Conceptualization; methodology; data curation. **Yalong An**: Methodology; software; visualization. **Ming Tian**: Methodology; investigation. **Xinmiao He**: Methodology; investigation. **Chengyi Song**: Methodology; software. **Jun He**: Conceptualization; methodology. **Xin Liu**: Methodology; investigation. **Ligang Wang**: Conceptualization; methodology; software. **Enrico D'Alessandro**: Methodology; resources. **Lixian Wang**: Funding acquisition; project administration; resources; supervision. **Yulong Yin**: Conceptualization; methodology; supervision; writing—review and editing. **Mingzhou Li**: Conceptualization; supervision; writing—review and editing. **Di Liu**: Conceptualization; supervision; resources; writing—review and editing. **Jinyong Wang**: Conceptualization; supervision; resources; funding acquisition; writing—review and editing. **Longchao Zhang**: Conceptualization; methodology; funding acquisition; project administration; supervision; resources; writing—review and editing.

## CONFLICT OF INTEREST STATEMENT

The authors declare no conflicts of interest.

## ETHICS STATEMENT

All the animals were handled in accordance with the “Guide for the Care and Use of Laboratory Animals” of the Institute of Animal Sciences, Chinese Academy of Agricultural Sciences (Beijing, China). All the procedures were approved by the Animal Care and Use Committee (IAS2022‐38). Pigs were euthanized according to the Animal Care Guidelines of the Ethics Committee of the Chinese Academy of Agricultural Sciences.

## Supporting information


**Figure S1**. Localized view of the gap in the T2T_RCpig1.0 genome.
**Figure S2**. Heatmaps of chromosomal interactions of T2T_Mpig1.0 and T2T_RCpig1.0 genomes.
**Figure S3**. Assessment of the genomic completeness of T2T_Mpig1.0 and T2T_RCpig1.0 using the BUSCO tool.
**Figure S4**. Heatmaps showing the distribution of telomeres in T2T_Mpig1.0 and T2T_RCpig1.0 genomes.
**Figure S5**. Total length of the Top ten repeat units in the genomes.
**Figure S6**. Characterization of repeating units in the centromere region of T2T_Mpig1.0.
**Figure S7**. Characterization of repeating units in the centromere region of T2T_RCpig1.0.
**Figure S8**. Presence and absence information of pan‐gene families in the 14 pig genomes.
**Figure S9**. Number of gene families in each category across individual genomes.
**Figure S10**. Comparative analysis of (A) FPKM, (B) CDS length, (C) Ka/Ks ratios, and (D) π values among core, dispensable, and private genes.
**Figure S11**. GO and KEGG enrichment analyses for core, dispensable, and private genes.
**Figure S12**. Geographic distribution of the pig breeds used in this study.
**Figure S13**. Pie chart showing the proportion of SVs identified through genome alignment and read mapping strategies.
**Figure S14**. Hourly measurements of temperature, humidity, and respiratory rate during heat stress in Rongchang pigs.
**Figure S15**. Volcano plots of differentially expressed genes (DEGs) under cold stress for Min pigs and heat stress for Rongchang pigs.
**Figure S16**. The missing of 89 DEGs reported in a previous study.
**Figure S17**. DEGs involved in GO terms and KEGG pathways under cold and heat exposures.
**Figure S18**. Assessment of cross‐correlation values (NSC and RSC) in heat stress and control samples.
**Figure S19**. Functional enrichment analysis of genes related to H3K27ac modification in skeletal muscle of Rongchang pigs from control and heat stress groups.
**Figure S20**. Validation of SV upstream of *GIMAP6* by electrophoresis.
**Figure S21**. Prediction of motifs in the DEL of the promoter region of *GIMAP6* and Top five GO terms.
**Figure S22**. Manhattan plots showing GWAS for six carcass traits based on SNPs and SVs.

**Table S1.** Summary of sequencing data for Min and Rongchang pigs.
**Table S2.** Statistics for T2T genome assemblies of the two pig breeds.
**Table S3.** Chromosome lengths of the T2T_Mpig1.0 and T2T_RCpig1.0 genomes.
**Table S4.** Statistics for the annotation of repetitive sequences.
**Table S5.** Statistics of gene structure annotation in the T2T_Mpig1.0 and T2T_RCpig1.0 genomes.
**Table S6**. Newly identified genes with gene symbol in the two T2T genomes.
**Table S7**. QVs for both T2T pig genomes.
**Table S8.** Functional annotation of predicted genes in the T2T_Mpig1.0 and T2T_RCpig1.0 genomes.
**Table S9.** Telomere statistics for the 100‐kb window at both ends of the T2T_Mpig1.0 and T2T_RCpig1.0 chromosomes.
**Table S10.** Centromeric regions of each chromosome in T2T_Mpig1.0 and T2T_RCpig1.0 genomes.
**Table S11.** GFF information from publicly available databases utilized in pan‐genome analysis.
**Table S12.** Distribution of pan‐genomic gene families.
**Table S13.** Distribution of the number of core, dispensable and private genes.
**Table S14.** Genome information for constructing a graph‐based pan‐genome.
**Table S15.** Short‐read data information for constructing a graph‐based pan‐genome.
**Table S16.** Short‐read data information based on graph‐based pan‐genome genotyping.
**Table S17.** KEGG enrichment analyses using cold and hot regions of pigs compared to Western commercial pig breeds.
**Table S18**. Sample information for heat stress experiments.
**Table S19.** Differential expression of genes in skeletal muscle of Min pigs after cold stress.
**Table S20.** Differential expression of genes in skeletal muscle of Rongchang pigs after heat stress.
**Table S21.** Differential peaks in skeletal muscle of Rongchang pigs after heat stress.

## Data Availability

Genome sequencing data are available in NCBI under the accession number PRJNA1180263 (https://www.ncbi.nlm.nih.gov/bioproject/PRJNA1180263). The genome assemblies of the Min and Rongchang pigs have been deposited in the NGDC Genome Warehouse under the accession numbers GWHFPUN00000000.1 (https://ngdc.cncb.ac.cn/gwh/Assembly/92156/show) and GWHFPUM00000000.1 (https://ngdc.cncb.ac.cn/gwh/Assembly/92155/show). The GFF annotation file is available at the following link: https://doi.org/10.6084/m9.figshare.28424744. The code and scripts used are stored in GitHub at https://github.com/zongzone/T2T_Pig. All data and materials were obtained from the corresponding authors. Supplementary materials (methods, figures, tables, slides, videos, Chinese translations, and updated materials) can be found online at DOI or iMeta Science http://www.imeta.science/. The data that support the findings of this study are openly available in Genome assembly of the Min and Rongchang pigs at https://www.ncbi.nlm.nih.gov/bioproject/PRJNA1180263, reference number PRJNA1180263.
