# Supplementary material for: Two telomere‐to‐telomere pig genome assemblies and pan‐genome analyses provide insights into genomic structural landscape and genetic adaptations
Source: Imeta. 2025 Apr 3;4(2):e70013. doi: 10.1002/imt2.70013 (PMC11995179; doi:10.1002/imt2.70013)
Supplement: Supplementary file 1 — Figure S1. Localized view of the gap in the T2T_RCpig1.0 genome. Figure S2. Heatmaps of chromosomal interactions of T2T_Mpig1.0 and T2T_RCpig1.0 genomes. Figure S3. Assessment of the genomic completeness of T2T_Mpig1.0 and T2T_RCpig1.0 using the BUSCO tool. Figure S4. Heatmaps showing the distribution of telomeres in T2T_Mpig1.0 and T2T_RCpig1.0 genomes. Figure S5. Total length of the Top ten repeat units in the genomes. Figure S6. Characterization of repeating units in the centromere region of T2T_Mpig1.0. Figure S7. Characterization of repeating units in the centromere region of T2T_RCpig1.0. Figure S8. Presence and absence information of pan‐gene families in the 14 pig genomes. Figure S9. Number of gene families in each category across individual genomes. Figure S10. Comparative analysis of (A) FPKM, (B) CDS length, (C) Ka/Ks ratios, and (D) π values among core, dispensable, and private genes. Figure S11. GO and KEGG enrichment analyses for core, dispensable, and private genes. Figure S12. Geographic distribution of the pig breeds used in this study. Figure S13. Pie chart showing the proportion of SVs identified through genome alignment and read mapping strategies. Figure S14. Hourly measurements of temperature, humidity, and respiratory rate during heat stress in Rongchang pigs. Figure S15. Volcano plots of differentially expressed genes (DEGs) under cold stress for Min pigs and heat stress for Rongchang pigs. Figure S16. The missing of 89 DEGs reported in a previous study. Figure S17. DEGs involved in GO terms and KEGG pathways under cold and heat exposures. Figure S18. Assessment of cross‐correlation values (NSC and RSC) in heat stress and control samples. Figure S19. Functional enrichment analysis of genes related to H3K27ac modification in skeletal muscle of Rongchang pigs from control and heat stress groups. Figure S20. Validation of SV upstream of GIMAP6 by electrophoresis. Figure S21. Prediction of motifs in the DEL of the promoter region of GIMAP6 and To [file IMT2-4-e70013-s001.docx]

**Supporting Infomation to:**

**Two telomere-to-telomere pig genome assemblies and pan-genome analyses provide insights into genomic structural landscape and genetic adaptations**

Wencheng Zong^1^, Li Chen^1,2^, Dongjie Zhang^3^, Yuebo Zhang^4,5^, Jinbu Wang^1^, Xinhua Hou^1^, Jie Chai^2^, Yalong An^6^, Ming Tian^3^, Xinmiao He^3^, Chengyi Song^7^, Jun He^4,5^, Xin Liu^1^ , Ligang Wang^1^, Enrico D’Alessandro^8^, Lixian Wang^1^, Yulong Yin^4,5^, Mingzhou Li^9^, Di Liu^3^, Jinyong Wang^2^, Longchao Zhang^1^

^1^ State Key Laboratory of Animal Biotech Breeding, Institute of Animal Sciences, Chinese Academy of Agricultural Sciences (CAAS), Beijing 100193, China

^2^ National Center of Technology Innovation for Pigs, Chongqing Academy of Animal Science, Chongqing 402460, China

^3^ Institute of Animal Husbandry, Heilongjiang Academy of Agricultural Sciences, Harbin, 150086, China

^4^ Yuelushan laboratory, Changsha, 410128, China

^5^ Key Laboratory of Livestock and Poultry Resources (Pig) Evaluation and Utilization, Ministry of Agriculture and Rural Affairs, College of Animal Science and Technology, Hunan Agricultural University, Changsha, China

^6^ Key Laboratory of Animal Genetics, Breeding and Reproduction of Shaanxi Province, College of Animal Science and Technology, Northwest A&F University, Xianyang 712100, China

^7^ College of Animal Science and Technology, Yangzhou University, Yangzhou 225009, China

^8^ Department of Veterinary Science, Division of Animal Production, University of Messina, Messina 98168, Italy

^9^ State Key Laboratory of Swine and Poultry Breeding Industry, College of Animal Science and Technology, Sichuan Agricultural University, Chengdu, 611130, China.

**Correspondence**

Longchao Zhang, State Key Laboratory of Animal Biotech Breeding, Institute of Animal Sciences, Chinese Academy of Agricultural Sciences (CAAS), Beijing 100193, China.

Email: [zhlchias@163.com](mailto:zhlchias@163.com)

Jinyong Wang, Chongqing Academy of Animal Science, Chongqing 402460, China.

Email: [kingyou@vip.sina.com](mailto:kingyou@vip.sina.com)

Di Liu, Institute of Animal Husbandry, Heilongjiang Academy of Agricultural Sciences, Harbin, 150086, China.

Email: [liudi1963@163.com](mailto:liudi1963@163.com)

Mingzhou Li, State Key Laboratory of Swine and Poultry Breeding Industry, College of Animal Science and Technology, Sichuan Agricultural University, Chengdu, 611130, China.

Email: [mingzhou.li@sicau.edu.cn](mailto:mingzhou.li@sicau.edu.cn)

Yulong Yin, Yuelushan laboratory, Changsha, 410128, China; Key Laboratory of Livestock and Poultry Resources (Pig) Evaluation and Utilization, Ministry of Agriculture and Rural Affairs, College of Animal Science and Technology, Hunan Agricultural University, Changsha, 410128, China.

Email: yinyulong@isa.ac.cn

Lixian Wang, State Key Laboratory of Animal Biotech Breeding, Institute of Animal Sciences, Chinese Academy of Agricultural Sciences (CAAS), Beijing 100193, China.

Email: iaswlx@263.net

Wencheng Zong, Li Chen, Dongjie Zhang, and Yuebo Zhang contributed equally to this study.

## **METHODS**

### **Sampling and sequencing**

Samples were collected from 6-month-old females of the Min pig from the Heilongjiang Academy of Agricultural Sciences (Harbin, Heilongjiang Province) and the Rongchang pig from the Chongqing Academy of Animal Science (Rongchang District, Chongqing). Min pigs inhabit the cold and arid Northeastern region of China, where average winter temperatures can drop to -20°C. In contrast, Rongchang pigs thrive in the hot and humid Southwestern region of China, with summer temperatures surpassing 38°C. The temperature data were retrieved from the China Meteorological Administration (<https://www.cma.gov.cn/>).

Total DNA was extracted from skeletal muscle tissue using a phenol-chloroform method. The concentration and quality of the DNA were assessed using a NANODROP 1000 spectrophotometer (Thermo Fisher Scientific, USA). PacBio HiFi libraries were constructed with an average insert size of 15 kb using SMRTbell Template Prep Kit and sequenced on a PacBio Sequel IIe platform. This yielded an approximate sequencing depth of 40× using PacBio HiFi technology. SMRTlink v6.0 (<https://www.pacb.com/>) was utilized to filter the HiFi data, retaining reads with quality scores above Q20 for subsequent analysis. Nanopore libraries were prepared using a Ligation Sequencing Kit (Oxford Nanopore Technologies, UK) and achieved an approximate depth of 140× on a PromethION 48 sequencer (Oxford Nanopore Technologies, UK). Guppy v6.1.5 [1] was employed to process the ONT data and retain reads with quality values above Q7. Illumina paired-end libraries with 350-bp insert sizes were constructed using the NEBNext Ultra DNA Library Prep Kit for Illumina (New England Biolabs, USA), followed by short-read sequencing. Approximately 50× next-generation sequencing was performed for the genome survey. Raw data were filtered using Fastp v0.23.1[2] to trim the ends of the reads, automatically identify suitable sequences and eliminate contamination. Hi-C libraries were prepared using a modified standard protocol [3] and sequenced on an Illumina NovaSeq platform to generate an approximate depth of 100×. Quality control was performed using HiCUP v0.5.9 [4] to remove duplicates and ensure accurate Hi-C read alignment. To improve the annotation quality, both ISO-seq and RNA-seq were performed. Total RNA was extracted from five tissues/organs (spleen, heart, skeletal muscle, adipose, and kidney) using TRIzol reagent (Invitrogen, USA) following the manufacturer’s instructions. RNA from each tissue/organ was pooled (5 µg total RNA) for Iso-Seq library construction, and sequenced on the PacBio Sequel IIe platform. RNA-seq libraries were prepared using the Illumina PE150 strategy, and Fastp v0.23.1 [2] was used to filter low-quality adapter sequences.

### **Genome survey, assembly, and assessment**

To estimate the complexity of the genome, the Kmer analysis method of Jellyfish v2.2.7 [5] was employed. This method provides information on genome size, heterozygosity, and the proportion of repetitive sequences in Min and Rongchang pigs.

Initially, genomes were assembled by integrating PacBio HiFi, ONT ( > 100 kb), and Hi-C reads in the T2T assembly mode using HiFiasm v.0.18.7 [6], with the parameters: --ul --h1 --h2 HiFireads. Hi-C sequencing data were used to anchor all contigs via the AllHiC pipeline [7,8] with the following parameters: --minREs 50, --maxlinkdensity 3, and --NonInformativeRatio 2. The contigs were then manually checked for orientation, and any misassemblies were corrected using Juicebox [9]. The ONT data were assembled using NextDenovo v2.5.0 [10] with the following setting: seed_cutoff = 75006. Subsequently, Illumina data and HiFi data were utilized to polish the ONT contigs with Nextpolish v1.4.0 [11] (parameters: lgs_options = -min_read_len 1k -max_read_len 100k -max_depth 50 sgs_options = -max_depth 200 -bwa), resulting in polished ONT assemblies. Gaps in the assembled genome were filled using two main approaches: 1) The first approach involved using ONT data to directly fill gaps in the chromosome-level assembly. This was achieved by utilizing TGS-gapcloser v1.2.1 [12], which used ONT data to bridge gaps in the assembly, ensuring more contiguous chromosome assemblies. 2) The second strategy employed MUMmer v4.0.0 [13] for the detection of gaps. Specifically, the ONT-based assembly data were mapped back to the chromosome-level assembly, and regions that required further refinement were identified.

To comprehensively assess genome quality, four assessment methods were employed: 1) Using Illumina and HiFi reads generated from the sequencing of two pig genomes to assess mapping and coverage ratios; 2) Employing BUSCO v5.7.1[14] with the mammalia_odb10 database to assess genome completeness; 3) Performing single nucleotide polymorphisms (SNPs) calling on assembled sequencing data using Genome Analysis Toolkit (GATK, v4.1.8.1) [15] to evaluate base accuracy; and 4) Determining the assembly base quality values (QVs) using Merqury v1.4.1 [16].

### **Genome annotation**

#### **Repeat annotation**

An integrated strategy combining homology matching and *de novo* searches was employed to identify genome-wide repetitive sequences in the repetitive annotation pipeline. Homology-based prediction utilized a pig transposable element (TE) database [17], previously annotated based on the RepBase database (http://www.girinst.org/repbase) and RepeatMasker (<http://www.repeatmasker.org/>) [18], to detect known repetitive sequences. For *de novo* prediction, a database of repetitive sequences was generated using RepeatModeler (http://www.repeatmasker.org/RepeatModeler.html) with the default settings. Subsequently, TE libraries containing homologous and *de novo* annotations were integrated for de-redundancy using UCLUST [19], retaining only full-length TE sequences to create non-redundant libraries that were provided to RepeatMasker to identify repeat sequences.

#### **Gene structure annotation**

Gene annotation was performed by combining homolog-based, transcriptome-based, and *ab initio* predictions. For the homolog-based approach, protein sequences from cattle (Bostau, *Bos taurus*), domestic dogs (Canfam, *Canis familiaris*), humans (Homsap, *Homo sapiens*), mice (Musmus, *Mus musculus*), sheep (Oviari, *Ovis aries*), and pigs (Susscr, *Sus scrofa*) were downloaded from NCBI or Ensembl. All protein sequences were aligned to genomic sequences using TblastN (v2.2.26; E-value ≤ 1e^-5^), and then sequences from BLAST hits were accurately spliced and compared using GeneWise v2.4.1 [20] to predict the gene structures contained in each protein region. For the transcriptome-based approach, RNA-seq data were first used for assembly using Trinity v2.8.5 [21]. The assembled transcripts were then merged with ISO-seq data to eliminate redundancy. The combined results were aligned to the genome to obtain gene structure information using Program to Assemble Spliced Alignments (PASA, v2.0.2) [22]. Additionally, RNA-seq data were aligned using Hisat2 v2.2.1 [23], while ISO-seq data were aligned using Minimap2 v2.2.8 [24]. Transcript predictions were subsequently performed using StringTie v2.1.6 [25]. For the *ab initio* prediction, gene prediction was performed using the automated gene prediction pipelines Augustus v3.5 [26] and SNAP (<http://korflab.ucdavis.edu/software.html>) [27]. Finally, the gene model evidence from these three methods was integrated with EVidenceModeler v1.1.1 [28] using PASA v2.0.2 [22] terminal exon support and masked TEs as inputs for gene prediction.

#### **Functional annotation**

Protein functions were predicted by transferring annotations from the closest BLAST hits (E-value < 1e^-5^) to the Swissprot (http://web.expasy.org/docs/swiss-prot_guideline.html) [29] and NR databases. Motifs and domains were annotated through InterProScan v5.39 [30] against several databases, including ProDom, PRINTS, Pfam, SMRT, PANTHER, and PROSITE. Each gene was assigned GO terms (http://www.geneontology.org/page/go-database) [31] based on the corresponding InterPro entries. Furthermore, gene sets with KEGG pathways (http://www.kegg.jp/kegg/kegg1.html) [32] were associated to identify the best matches for each gene (E-value < 1e^-5^).

#### **Non-coding RNA annotation**

The tRNAs were identified using the tRNAscan-SE v1.4 [33] (http://lowelab.ucsc.edu/tRNAscan-SE/). Given the high inter-species conservation of rRNAs, rRNA sequences were used from related species as references and the rRNA sequences were predicted using BLAST v2.2.26. Furthermore, non-coding RNAs (ncRNAs), such as miRNAs and snRNAs, were discovered by querying the Rfam database [34] with default parameters using Infernal software (<http://infernal.janelia.org/>).

### **Annotation of previously unresolved genomic regions and new genes**

Sscrofa11.1 was compared to the T2T_Mpig1.0 genome using MUMmer v4.0.0 [13] to identify previously unresolved regions (PURs). All uncovered regions were classified as PURs. Subsequently, we annotated these regions using RepeatMasker [18].

For newly annotated genes, we followed two approaches. First, we identified the coding sequences within the PURs. Second, we extracted the genomic sequences of all genes from the T2T genomes and aligned them with Sscrofa11.1 using MMseqs2 v16.747c6 [35] (parameters: easy-search --search-type 3). Genes with sequence identity below 80% and coverage below 50% were retained. Finally, the genes identified by both methods were subjected to de-redundancy processing to eliminate duplicate entries, and all genes were then validated against the Ensembl database (https://www.ensembl.org/Sus_scrofa/Info/Index).

### **Identification of telomeres and centromeres**

Telomere sequence identification was performed using Tidk v.0.2.0 (<https://github.com/tolkit/telomeric-identifier>) with a 100-kb window for scanning. A telomere was considered present if the number of telomeric repeat sequences exceeded 50 copies within a 100-kb region at chromosome ends.

Centromeric regions were identified by detecting the genome with tandem repeats (TRs) using the Tandem Repeats Finder (TRF, v4.09) [36] with the parameters 2 7 7 80 10 50 2000 -f -d -m -l 15. Subsequently, the results were formatted using TRF2GFF (https://github.com/Adamtaranto/TRF2GFF). Finally, coding gene and TE annotations were integrated to accurately determine centromeric regions using the Integrative Genomics Viewer (IGV) [37].

### **Pan-genome construction**

To construct a comprehensive pan-genome based on coding genes, gene annotations for publicly available pig breeds, including Bamei, Berkshire, Duroc, Hampshire, Jinhua, Landrace, Large White, Meishan, Pietrain, Tibetan, Cross (Usmarc), and Wuzhishan, were collected. For each breed, only the longest transcript was retained, and genes encoding proteins of fewer than 50 amino acids were excluded. An all-against-all comparison was performed using BlastP v2.9.0+ with an E-value of 1e^-5^. Gene family clustering was performed using OrthoFinder v2.5.5 [38]. Based on the clustering results, gene families common to all genomes were defined as core gene families, those present in genomes 2–13 as dispensable gene families, and those present in only one genome as private gene families. The non-synonymous to synonymous substitution ratio (Ka/Ks) for each gene in the pan-genome was calculated with WGD v1.1.2 [39], using output from OrthoFinder. Multiple sequence alignments were performed with MAFFT v7.475 [40] to calculate nucleotide diversity (π) for each gene in the pan-genome. The π calculation formula is: $\pi= \frac{D}{L\times\frac{N\times(N-1)}{2}}$, where *D* is the number of differentiated sites, *L* is the conserved alignment length, and *N* is the number of sequences [41].

### **SV identification and graph pan-genome construction**

The gap-free T2T_Mpig1.0 genome was employed as a reference genome for alignment with the other 12 genomes, including T2T_RCpig1.0, Ossabaw, Duroc (Sscrofa11.1), Babraham, Cross (Usmarc), Nero Siciliano, Bama, Bamei, Luchuan, Ningxiang, Meishan, and Nanchukmacdon, using MUMmer v4.0.0 [13]. Two sets of structural variation (SV) calls were generated using SVMU[42] and SyRI v1.6.3 [43], respectively, both with default parameters. For SV detection with SVMU [42], the results were used to produce the deletion (DEL) and insertion (INS). DEL and INS greater than 50 bp in homologous comparison regions were retained. For SV calls using SyRI v1.6.3 [43], SVs with DEL, INS, inversion (INV), and translocation (TRA) greater than 50 bp were retained. SVs containing “N” sequences were removed. The set of SVs, called by SVMU [42] and SYRI v1.6.3 [43], was then merged with the identified SVs in each sample using SURVIVOR v.1.0.6 [44] with the following parameters: 50 1 0 0 0 0.

Additionally, to obtain more comprehensive variation information, at least 30× short-read sequencing of 5 Nero Siciliano pigs from Italy was performed. Data from NCBI and NGDC were collected on five Rongchang pigs, five Duroc pigs, five Bamei pigs, five Crossbred pigs, five Nanchukmacdon pigs, five Min pigs, five Ossabaw pigs, two Babraham pigs, five Ningxiang pigs, five Bama pigs, five Luchuan pigs, and five Meishan pigs. The re-sequencing data were first mapped to the T2T_Mpig1.0 genome using BWA v0.7.17-r1188 [45]. The generated alignment files were then sorted using SAMtools v1.18 [46], followed by duplicate marking with Picard v3.3.0 [47]. SV calling and merging were performed using Smoove v0.2.8 (https://github.com/brentp/smoove) with default parameters. Considering the limitations of short-read sequencing for SV identification, only SVs ranging from 50 bp to 1 Mb were retained.

The results from the genome alignment and short-read data identification were combined, considering variants with more than 75% overlap to be the same. The genome-aligned variants were preferentially retained. Then, T2T_Mpig1.0 genome was used as a reference genome to construct all presence/absence variations (PAVs) into graph pan-genome based on vg [48]. For population-scale studies of SVs, we downloaded 246 high-depth (~ 20×) Illumina short reads from pigs and mapped to a graph pan-genome for genotyping using the vg [48] with default parameters.

### **Population genetic structure analysis**

All SVs were initially filtered with PLINK v1.90 [49] using the following parameters: geno 0.1, maf 0.05, and --chr 1-18. Principal component analysis (PCA) was conducted using GCTA v1.94.1 [50], with the first two principal components used for plotting. Phylogenetic analysis was performed by calculating *p*-distances in VCF2Dis v1.53 (https://github.com/BGI-shenzhen/VCF2Dis), followed by the construction of neighbor-joining trees with FastME 2.0 (www.atgc-montpellier.fr/fastme/) [51]. The population structure was analyzed using Admixture v1.3.0 [52] with K values of 3 and 35.

### **Detection of candidate population stratification signals**

To identify candidate SVs linked to environmental adaptation, the DI_SV_ method was applied, which highlights the differences in allele frequencies between populations [53]. Pig breeds located in temperate continental, monsoon, and highland climates are classified as cold region breeds, whereas those in subtropical and tropical monsoon climates are categorized as hot region breeds. We compared 112 hot and 72 cold region Chinese pig breeds with Western commercial pig breeds, and identified the top 5% of SVs as candidate population-stratified loci.

### **Temperature stress experiment design**

For the cold-adapted group, we downloaded data from a previous cold stress experiment on Min pigs [54].

For the heat-adapted group, a heat stress experiment was conducted on Rongchang pigs in a temperature-controlled room in Rongchang District, Chongqing, China. Six castrated male pigs, each approximately 90-day-old, were selected and randomly divided into two groups: three pigs were assigned to the experimental group, and three to the control group. Details of specific samples are provided in Table S18. The temperature was maintained at 38±1°C for the experimental group and 25±1°C for the control group. To minimize experimental errors, the pigs were acclimatized in a temperature-controlled room for one week prior to the experiment. During this acclimation period, the pigs were given free access to food and water. Subsequently, three pigs from the control group were sacrificed before the temperature adjustment. Twenty-four hours later, the temperature of the temperature-controlled room was adjusted to 38°C to simulate typical summer conditions in Chongqing. During this process, it was observed that the humidity fluctuated in response to the temperature changes, and the respiration rate of the pigs continuously increased. Once the respiration rate had stabilized for 5 hours, the three heat-stressed pigs were slaughtered. All tissues were immediately stored in liquid nitrogen after collection. Skeletal muscle tissues were selected for RNA sequencing and CUT&Tag sequencing.

### **RNA-seq library construction, sequencing, and analysis**

Total RNA was isolated using the TRIzol reagent (Invitrogen, USA) following the manufacturer’s protocol. The primary criterion for assessing the quality of RNA samples was an RNA integrity number of 7 or higher. RNA-seq libraries were prepared using the NEBNext Ultra Directional RNA Library Prep Kit for Illumina (New England Biolabs, USA) and sequenced on the Illumina NovaSeq 6000 platform.

In the data-processing stage, adapters and low-quality sequences were trimmed from the raw data using Fastp v0.23.1 [2]. The clean reads were then mapped to the T2T_Mpig1.0 genome using Hisat2 v2.2.1 [23] with default parameters. The resulting alignment files were converted to the BAM format using SAMtools v1.18 [46]. Read counts for each gene were calculated from the BAM files using StringTie v2.2.1 [25]. Gene expression levels were normalized to Fragments Per Kilobase of transcript per Million mapped reads (FPKM) values through an internal script based on the read count table. Differential gene expression analysis was conducted using the DESeq2 package [55] in R, with the thresholds as |log2FoldChange| > 0.59 and *p*agj < 0.05.

### **CUT&Tag library construction, sequencing, and analysis**

An H3K27ac (Abcam Cat# ab4729)-labeled CUT&Tag assay was carried out to conduct an analysis of chromatin epigenomics, following a previously established protocol [56]. The DNA sequencing was performed using an Illumina NovaSeq platform at Wuhan Frasergen Bioinformatics Co., Ltd.

In the process of CUT&Tag analysis, the original raw data underwent quality control using Fastp v0.23.1 [2]. After quality control, the clean data were aligned with the T2T_Mpig1.0 genome using Bowtie2 v2.4.5 [57]. The reads from the alignment were further sorted and PCR duplicates were eliminated using SAMtools v1.18 [46] and Picard v3.3.0 [47], thereby obtaining the final valid data for subsequent analysis. Peak calling was performed on the final valid data using Macs2 [58] with the parameters of -q 0.05 --nomodel --extsize 150 -B --keep-dup=all --call-summits. DESeq2 package [55] in R was used to perform between-group differential peak (DP) analysis. Regions with analysis results satisfying |log2FoldChange| > 0.59 and *p* < 0.05 were designated as significant DP. Additionally, motifs in the DEL sequence were identified using MEME v5.5.7 [59] and the GO terms of the motifs were enriched using GOMo v5.5.7 [60].

### **Primer design and PCR amplification**

Primers utilized in this study were designed based on the sequences that extended approximately 300 bp both upstream and downstream of the DEL in the *GIMAP6* gene. The primer sequences are as follows: the forward primer is TGTTCCCAGGAAGTCTACCACG and the reverse primer is GGGTGCATGCATCTCTTGAAT. The PCR amplification conditions included an initial denaturation at 95°C for 3 minutes, followed by 30 cycles consisting of denaturation at 95°C for 15 seconds, annealing at 58°C for 15 seconds, and extension at 72°C for 1 minute, with a final extension at 72°C for 5 minutes to ensure complete amplification.

### **SNP and SV calling**

For SNPs, the re-sequencing data obtained from 540 individuals of the Large White × Min pig resource population were initially aligned to the T2T_Mpig1.0 genome using BWA v0.7.17-r1188 [45]. The resulting alignment files were processed through SAMtools v1.18 [46] for coordinate-based sorting, followed by duplicate marking using Picard v3.3.0 [47]. SNPs were detected using the GATK v4.1.8.1 [15] with the “HaplotypeCaller,” “GenotypeGVCFs,” and “SelectVariants” tools. After calling, “VariantFiltration” was applied using the following parameters: QD < 2.0, FS > 60.0, MQ < 40.0, MQRankSum < -12.5, ReadPosRankSum < -8.0, and SOR > 3.0.

For SVs, the re-sequencing dataset was genotyped by mapping to a graph pan-genome using vg [48].

### **GWAS and GS**

To systematically evaluate the impact of SVs on the resolution of genome-wide association studies (GWAS) and the accuracy of genomic selection (GS), we selected six representative carcass traits, namely slaughter weight (measured at 240 days), oblique carcass length, straight carcass length, cannon circumference, body height, and hip circumference, for subsequent analysis.

For GWAS, we performed quality control on all markers (PLINK: --maf 0.05 --geno 0.1), retaining 73,627 high-quality SVs and 17,636,239 high-quality SNPs. EMMAX[61] employs mixed linear models for GWAS of SNPs and SVs. Sex and slaughter batches were treated as fixed effects, and PCA results were included as covariates. The significance threshold was set at 5 × 10⁻⁸, which is the widely accepted gold standard for GWAS [62]. All the GWAS results were visualized using the rMVP package [63].

For GS, we filtered SNPs with high LD (PLINK --indep-pairwise 1000 5 0.5 parameters) to ensure efficient processing. As a result, the SNP and SNP+SV panels contained 1,083,305 and 1,134,860 markers, respectively. We calculated the feature gain of each marker using the LightGBM algorithm for three marker panels (SNP, SNP+SV, and SV) and selected the markers with positive gains for subsequent genome prediction. LightGBM and GBLUP were implemented in R using the LightGBM [64] and rrBLUP [65] packages, respectively. The prediction accuracy of the model was evaluated by calculating the Pearson correlation between the true phenotypes and the predicted GEBVs. The final results were obtained through 10-fold cross-validation with 10 replications.

## **REFERENCES**

1. Sherathiya, Venus N., Michael D. Schaid, Jillian L. Seiler, Gabriela C. Lopez, Talia N. Lerner. 2021. “GuPPy, a Python toolbox for the analysis of fiber photometry data.” *Scientific Reports* 11: 24212. <https://doi.org/10.1038/s41598-021-03626-9>

2. Chen, Shifu, Yanqing Zhou, Yaru Chen, Jia Gu. 2018. “fastp: an ultra-fast all-in-one FASTQ preprocessor.” *Bioinformatics* 34: i884−i890. <https://doi.org/10.1093/bioinformatics/bty560>

3. Belton, Jon-Matthew, Rachel Patton McCord, Johan Harmen Gibcus, Natalia Naumova, Ye Zhan, Job Dekker. 2012. “Hi–C: A comprehensive technique to capture the conformation of genomes.” *Methods* 58: 268-276. <https://doi.org/https://doi.org/10.1016/j.ymeth.2012.05.001>

4. Wingett, S., P. Ewels, M. Furlan-Magaril, T. Nagano, S. Schoenfelder, P. Fraser, S. Andrews. 2015. “HiCUP: pipeline for mapping and processing Hi-C data.” *F1000Research* 4: 1310.

5. Marçais, Guillaume, Carl Kingsford. 2011. “A fast, lock-free approach for efficient parallel counting of occurrences of k-mers.” *Bioinformatics* 27: 764−770. <https://doi.org/10.1093/bioinformatics/btr011>

6. Cheng, Haoyu, Gregory T. Concepcion, Xiaowen Feng, Haowen Zhang, Heng Li. 2021. “Haplotype-resolved de novo assembly using phased assembly graphs with hifiasm.” *Nature Methods* 18: 170−175. <https://doi.org/10.1038/s41592-020-01056-5>

7. Zhang, Jisen, Xingtan Zhang, Haibao Tang, Qing Zhang, Xiuting Hua, Xiaokai Ma, Fan Zhu, et al. 2018. “Allele-defined genome of the autopolyploid sugarcane Saccharum spontaneum L.” *Nature Genetics* 50: 1565−1573. <https://doi.org/10.1038/s41588-018-0237-2>

8. Zhang, Xingtan, Shengcheng Zhang, Qian Zhao, Ray Ming, Haibao Tang. 2019. “Assembly of allele-aware, chromosomal-scale autopolyploid genomes based on Hi-C data.” *Nature Plants* 5: 833−845. <https://doi.org/10.1038/s41477-019-0487-8>

9. Robinson, James T., Douglass Turner, Neva C. Durand, Helga Thorvaldsdóttir, Jill P. Mesirov, Erez Lieberman Aiden. 2018. “Juicebox.js Provides a Cloud-Based Visualization System for Hi-C Data.” *Cell Systems* 6: 256−258.e251. <https://doi.org/https://doi.org/10.1016/j.cels.2018.01.001>

10. Hu, Jiang, Zhuo Wang, Zongyi Sun, Benxia Hu, Adeola Oluwakemi Ayoola, Fan Liang, Jingjing Li, et al. 2024. “NextDenovo: an efficient error correction and accurate assembly tool for noisy long reads.” *Genome Biology* 25: 107. <https://doi.org/10.1186/s13059-024-03252-4>

11. Hu, Jiang, Junpeng Fan, Zongyi Sun, Shanlin Liu. 2020. “NextPolish: a fast and efficient genome polishing tool for long-read assembly.” *Bioinformatics* 36: 2253−2255. <https://doi.org/10.1093/bioinformatics/btz891>

12. Xu, Mengyang, Lidong Guo, Shengqiang Gu, Ou Wang, Rui Zhang, Brock A. Peters, Guangyi Fan, et al. 2020. “TGS-GapCloser: A fast and accurate gap closer for large genomes with low coverage of error-prone long reads.” *GigaScience* 9: giaa094. <https://doi.org/10.1093/gigascience/giaa094>

13. Marçais, G. Auid-Orcid, A. L. Delcher, A. M. Phillippy, R. Coston, S. L. Salzberg, A. Auid-Orcid Zimin. 2018. “MUMmer4: A fast and versatile genome alignment system.” *PLOS Computational Biology* 14: e1005944. <https://journals.plos.org/ploscompbiol/article/file?id=10.1371/journal.pcbi.1005944&type=printable>

14. Manni, Mosè, Matthew R. Berkeley, Mathieu Seppey, Evgeny M. Zdobnov. 2021. “BUSCO: Assessing Genomic Data Quality and Beyond.” *Current Protocols* 1: e323. <https://doi.org/https://doi.org/10.1002/cpz1.323>

15. McKenna, A., Eric Hanna M Fau - Banks, Andrey Banks E Fau - Sivachenko, Kristian Sivachenko A Fau - Cibulskis, Andrew Cibulskis K Fau - Kernytsky, Kiran Kernytsky A Fau - Garimella, David Garimella K Fau - Altshuler, et al. 2010. “The Genome Analysis Toolkit: a MapReduce framework for analyzing next-generation DNA sequencing data.” *Genome research* 20: 1297−1303.

16. Rhie, Arang, Brian P. Walenz, Sergey Koren, Adam M. Phillippy. 2020. “Merqury: reference-free quality, completeness, and phasing assessment for genome assemblies.” *Genome Biology* 21: 245. <https://doi.org/10.1186/s13059-020-02134-9>

17. Chen, Cai, Wei Wang, Xiaoyan Wang, Dan Shen, Saisai Wang, Yali Wang, Bo Gao, et al. 2019. “Retrotransposons evolution and impact on lncRNA and protein coding genes in pigs.” *Mobile DNA* 10: 19. <https://doi.org/10.1186/s13100-019-0161-8>

18. Tarailo-Graovac, Maja, Nansheng Chen. 2009. “Using RepeatMasker to Identify Repetitive Elements in Genomic Sequences.” *Current Protocols in Bioinformatics* 25: 4.10.11−14.10.14. <https://doi.org/https://doi.org/10.1002/0471250953.bi0410s25>

19. Edgar, Robert C. 2010. “Search and clustering orders of magnitude faster than BLAST.” *Bioinformatics* 26: 2460−2461. <https://doi.org/10.1093/bioinformatics/btq461>

20. Birney, E., Richard Clamp M Fau - Durbin, R. Durbin. 2004. “GeneWise and Genomewise.” *Genome Rsearch* 14: 988−995.

21. Grabherr, Manfred G., Brian J. Haas, Moran Yassour, Joshua Z. Levin, Dawn A. Thompson, Ido Amit, Xian Adiconis, et al. 2011. “Full-length transcriptome assembly from RNA-Seq data without a reference genome.” *Nature Biotechnology* 29: 644−652. <https://doi.org/10.1038/nbt.1883>

22. Haas, Brian J., Arthur L. Delcher, Stephen M. Mount, Jennifer R. Wortman, Roger K. Smith Jr, Linda I. Hannick, Rama Maiti, et al. 2003. “Improving the Arabidopsis genome annotation using maximal transcript alignment assemblies.” *Nucleic Acids Research* 31: 5654−5666. <https://doi.org/10.1093/nar/gkg770>

23. Kim, Daehwan, Ben Langmead, Steven L. Salzberg. 2015. “HISAT: a fast spliced aligner with low memory requirements.” *Nature Methods* 12: 357−360. <https://doi.org/10.1038/nmeth.3317>

24. Li, H. 2018. “Minimap2: pairwise alignment for nucleotide sequences.” *Bioinformatics* 34: 3094−3100.

25. Pertea, Mihaela, Geo M. Pertea, Corina M. Antonescu, Tsung-Cheng Chang, Joshua T. Mendell, Steven L. Salzberg. 2015. “StringTie enables improved reconstruction of a transcriptome from RNA-seq reads.” *Nature Biotechnology* 33: 290−295. <https://doi.org/10.1038/nbt.3122>

26. Stanke, Mario, Oliver Keller, Irfan Gunduz, Alec Hayes, Stephan Waack, Burkhard Morgenstern. 2006. “AUGUSTUS: ab initio prediction of alternative transcripts.” *Nucleic Acids Research* 34: W435−W439. <https://doi.org/10.1093/nar/gkl200>

27. Korf, Ian. 2004. “Gene finding in novel genomes.” *BMC Bioinformatics* 5: 59. <https://doi.org/10.1186/1471-2105-5-59>

28. Haas, Brian J., Steven L. Salzberg, Wei Zhu, Mihaela Pertea, Jonathan E. Allen, Joshua Orvis, Owen White, C. Robin Buell, Jennifer R. Wortman. 2008. “Automated eukaryotic gene structure annotation using EVidenceModeler and the Program to Assemble Spliced Alignments.” *Genome Biology* 9: R7. <https://doi.org/10.1186/gb-2008-9-1-r7>

29. Bairoch, Amos, Rolf Apweiler. 2000. “The SWISS-PROT protein sequence database and its supplement TrEMBL in 2000.” *Nucleic Acids Research* 28: 45−48. <https://doi.org/10.1093/nar/28.1.45>

30. Jones, Philip, David Binns, Hsin-Yu Chang, Matthew Fraser, Weizhong Li, Craig McAnulla, Hamish McWilliam, et al. 2014. “InterProScan 5: genome-scale protein function classification.” *Bioinformatics* 30: 1236-1240. <https://doi.org/10.1093/bioinformatics/btu031>

31. Gene Ontology, Consortium. 2004. “The Gene Ontology (GO) database and informatics resource.” *Nucleic Acids Research* 32: D258−D261. <https://doi.org/10.1093/nar/gkh036>

32. Kanehisa, M. 2002. “The KEGG database.” 247: 91-101.

33. Lowe, Todd M., Sean R. Eddy. 1997. “tRNAscan-SE: A Program for Improved Detection of Transfer RNA Genes in Genomic Sequence.” *Nucleic Acids Research* 25: 955−964. <https://doi.org/10.1093/nar/25.5.955>

34. Kalvari, Ioanna, Eric P. Nawrocki, Joanna Argasinska, Natalia Quinones-Olvera, Robert D. Finn, Alex Bateman, Anton I. Petrov. 2018. “Non-Coding RNA Analysis Using the Rfam Database.” *Current Protocols in Bioinformatics* 62: e51. <https://doi.org/https://doi.org/10.1002/cpbi.51>

35. Steinegger, Martin, Johannes Söding. 2017. “MMseqs2 enables sensitive protein sequence searching for the analysis of massive data sets.” *Nature Biotechnology* 35: 1026−1028. <https://doi.org/10.1038/nbt.3988>

36. Benson, Gary. 1999. “Tandem repeats finder: a program to analyze DNA sequences.” *Nucleic Acids Research* 27: 573−580. <https://doi.org/10.1093/nar/27.2.573>

37. Thorvaldsdóttir, Helga, James T. Robinson, Jill P. Mesirov. 2013. “Integrative Genomics Viewer (IGV): high-performance genomics data visualization and exploration.” *Briefings in Bioinformatics* 14: 178−192. <https://doi.org/10.1093/bib/bbs017>

38. Emms, David M., Steven Kelly. 2019. “OrthoFinder: phylogenetic orthology inference for comparative genomics.” *Genome Biology* 20: 238. <https://doi.org/10.1186/s13059-019-1832-y>

39. Chen, Hengchi, Arthur Zwaenepoel. 2023. Inference of Ancient Polyploidy from Genomic Data. *Methods in Molecular Biology* 2545: 3−18. <https://doi.org/10.1007/978-1-0716-2561-3_1>

40. Katoh, Kazutaka, Kazuharu Misawa, Kei‐ichi Kuma, Takashi Miyata. 2002. “MAFFT: a novel method for rapid multiple sequence alignment based on fast Fourier transform.” *Nucleic Acids Research* 30: 3059−3066. <https://doi.org/10.1093/nar/gkf436>

41. Chen, Jinfeng, Yang Liu, Minxuan Liu, Wenlei Guo, Yongqiang Wang, Qiang He, Weiyao Chen, et al. 2023. “Pangenome analysis reveals genomic variations associated with domestication traits in broomcorn millet.” *Nature Genetics* 55: 2243−2254. <https://doi.org/10.1038/s41588-023-01571-z>

42. Chakraborty, Mahul, J. J. Emerson, Stuart J. Macdonald, Anthony D. Long. 2019. “Structural variants exhibit widespread allelic heterogeneity and shape variation in complex traits.” *Nature Communications* 10: 4872. <https://doi.org/10.1038/s41467-019-12884-1>

43. Goel, Manish, Hequan Sun, Wen-Biao Jiao, Korbinian Schneeberger. 2019. “SyRI: finding genomic rearrangements and local sequence differences from whole-genome assemblies.” *Genome Biology* 20: 277. <https://doi.org/10.1186/s13059-019-1911-0>

44. Jeffares, Daniel C., Clemency Jolly, Mimoza Hoti, Doug Speed, Liam Shaw, Charalampos Rallis, Francois Balloux, Christophe Dessimoz, Jürg Bähler, Fritz J. Sedlazeck. 2017. “Transient structural variations have strong effects on quantitative traits and reproductive isolation in fission yeast.” *Nature Communications* 8: 14061. <https://doi.org/10.1038/ncomms14061>

45. Li, Heng, Richard Durbin. 2009. “Fast and accurate short read alignment with Burrows–Wheeler transform.” *Bioinformatics* 25: 1754−1760. <https://doi.org/10.1093/bioinformatics/btp324>

46. Li, Heng, Bob Handsaker, Alec Wysoker, Tim Fennell, Jue Ruan, Nils Homer, Gabor Marth, Goncalo Abecasis, Richard Durbin, Subgroup Genome Project Data Processing. 2009. “The Sequence Alignment/Map format and SAMtools.” *Bioinformatics* 25: 2078−2079. <https://doi.org/10.1093/bioinformatics/btp352>

47. Broad, Institute. 2019. 'Picard toolkit'.

48. Garrison, Erik, Jouni Sirén, Adam M. Novak, Glenn Hickey, Jordan M. Eizenga, Eric T. Dawson, William Jones, et al. 2018. “Variation graph toolkit improves read mapping by representing genetic variation in the reference.” *Nature Biotechnology* 36: 875-879. <https://doi.org/10.1038/nbt.4227>

49. Purcell, Shaun, Benjamin Neale, Kathe Todd-Brown, Lori Thomas, Manuel A. R. Ferreira, David Bender, Julian Maller, et al. 2007. “PLINK: A Tool Set for Whole-Genome Association and Population-Based Linkage Analyses.” *The American Journal of Human Genetics* 81: 559−575. <https://doi.org/10.1086/519795>

50. Yang, Jian, S. Hong Lee, Michael E. Goddard, Peter M. Visscher. 2011. “GCTA: A Tool for Genome-wide Complex Trait Analysis.” *The American Journal of Human Genetics* 88: 76−82. <https://doi.org/10.1016/j.ajhg.2010.11.011>

51. Lefort, Vincent, Richard Desper, Olivier Gascuel. 2015. “FastME 2.0: A Comprehensive, Accurate, and Fast Distance-Based Phylogeny Inference Program.” *Molecular Biology and Evolution* 32: 2798−2800. <https://doi.org/10.1093/molbev/msv150>

52. Alexander, D. H., Kenneth Novembre J Fau - Lange, K. Lange. 2009. “Fast model-based estimation of ancestry in unrelated individuals.” *Genome research* 19: 1655−1664. <https://doi.org/doi>: 10.1101/gr.094052.109

53. Cumer, Tristan, Frédéric Boyer, François Pompanon. 2021. “Genome-Wide Detection of Structural Variations Reveals New Regions Associated with Domestication in Small Ruminants.” *Genome Biology and Evolution* 13: evab165. <https://doi.org/10.1093/gbe/evab165>

54. Zhang, Dongjie, Shouzheng Ma, Liang Wang, Hong Ma, Wentao Wang, Jiqao Xia, Di Liu. 2022. “Min pig skeletal muscle response to cold stress.” *PLoS ONE* 17: e0274184. <https://doi.org/10.1371/journal.pone.0274184>

55. Love, Michael I., Wolfgang Huber, Simon Anders. 2014. “Moderated estimation of fold change and dispersion for RNA-seq data with DESeq2.” *Genome Biology* 15: 550. <https://doi.org/10.1186/s13059-014-0550-8>

56. Kaya-Okur, Hatice S., Steven J. Wu, Christine A. Codomo, Erica S. Pledger, Terri D. Bryson, Jorja G. Henikoff, Kami Ahmad, Steven Henikoff. 2019. “CUT&Tag for efficient epigenomic profiling of small samples and single cells.” *Nature Communications* 10: 1930. <https://doi.org/10.1038/s41467-019-09982-5>

57. Langmead, Ben, Steven L. Salzberg. 2012. “Fast gapped-read alignment with Bowtie 2.” *Nature Methods* 9: 357−359. <https://doi.org/10.1038/nmeth.1923>

58. Feng, Jianxing, Tao Liu, Bo Qin, Yong Zhang, Xiaole Shirley Liu. 2012. “Identifying ChIP-seq enrichment using MACS.” *Nature Protocols* 7: 1728−1740. <https://doi.org/10.1038/nprot.2012.101>

59. Bailey, Timothy L., James Johnson, Charles E. Grant, William S. Noble. 2015. “The MEME Suite.” *Nucleic Acids Research* 43: W39−W49. <https://doi.org/10.1093/nar/gkv416>

60. Buske, Fabian A., Mikael Bodén, Denis C. Bauer, Timothy L. Bailey. 2010. “Assigning roles to DNA regulatory motifs using comparative genomics.” *Bioinformatics* 26: 860−866. <https://doi.org/10.1093/bioinformatics/btq049>

61. Kang, Hyun Min, Jae Hoon Sul, Susan K. Service, Noah A. Zaitlen, Sit-yee Kong, Nelson B. Freimer, Chiara Sabatti, Eleazar Eskin. 2010. “Variance component model to account for sample structure in genome-wide association studies.” *Nature Genetics* 42: 348−354. <https://doi.org/10.1038/ng.548>

62. Jannot, Anne-Sophie, Georg Ehret, Thomas Perneger. 2015. “P < 5 × 10−8 has emerged as a standard of statistical significance for genome-wide association studies.” *Journal of Clinical Epidemiology* 68: 460−465. <https://doi.org/https://doi.org/10.1016/j.jclinepi.2015.01.001>

63. Yin, Lilin, Haohao Zhang, Zhenshuang Tang, Jingya Xu, Dong Yin, Zhiwu Zhang, Xiaohui Yuan, et al. 2021. “rMVP: A Memory-Efficient, Visualization-Enhanced, and Parallel-Accelerated Tool for Genome-Wide Association Study.” *Genomics, Proteomics & Bioinformatics* 19: 619−628. <https://doi.org/10.1016/j.gpb.2020.10.007>

64. Ke, Guolin, Qi Meng, Thomas Finley, Taifeng Wang, Wei Chen, Weidong Ma, Qiwei Ye, Tie-Yan Liu. 2017. 'LightGBM: a highly efficient gradient boosting decision tree', *Proceedings of the 31st International Conference on Neural Information Processing Systems*, Curran Associates Inc., Long Beach, California, USA, pp. 3149–3157.

65. Endelman, Jeffrey B. 2011. “Ridge Regression and Other Kernels for Genomic Selection with R Package rrBLUP.” *The Plant Genome* 4: 250−255. <https://doi.org/https://doi.org/10.3835/plantgenome2011.08.0024>


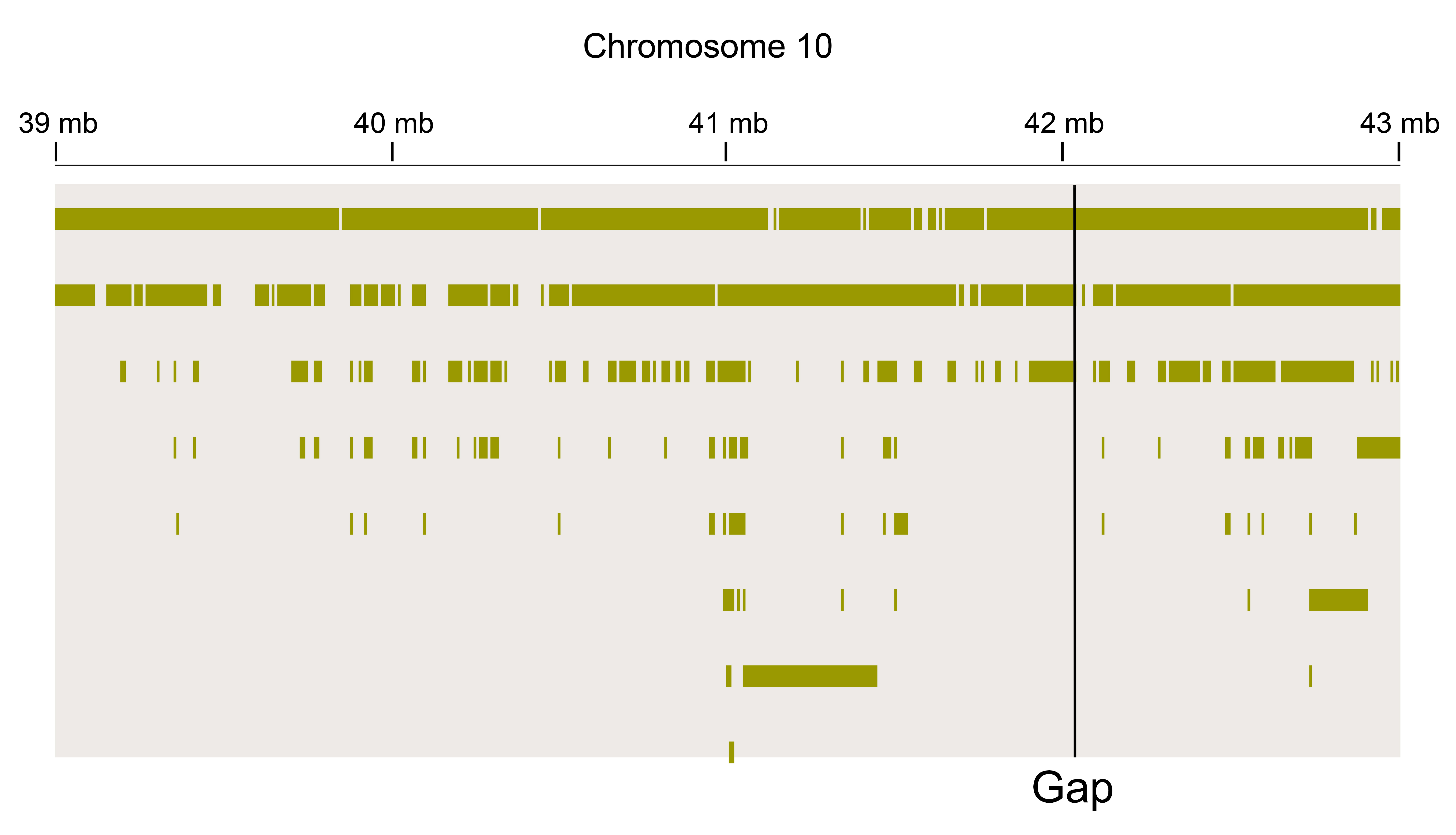


**Figure S1. Localized view of the gap in the T2T_RCpig1.0 genome.** Rectangles indicate repetitive sequences, while the black line marks the location of the gap.


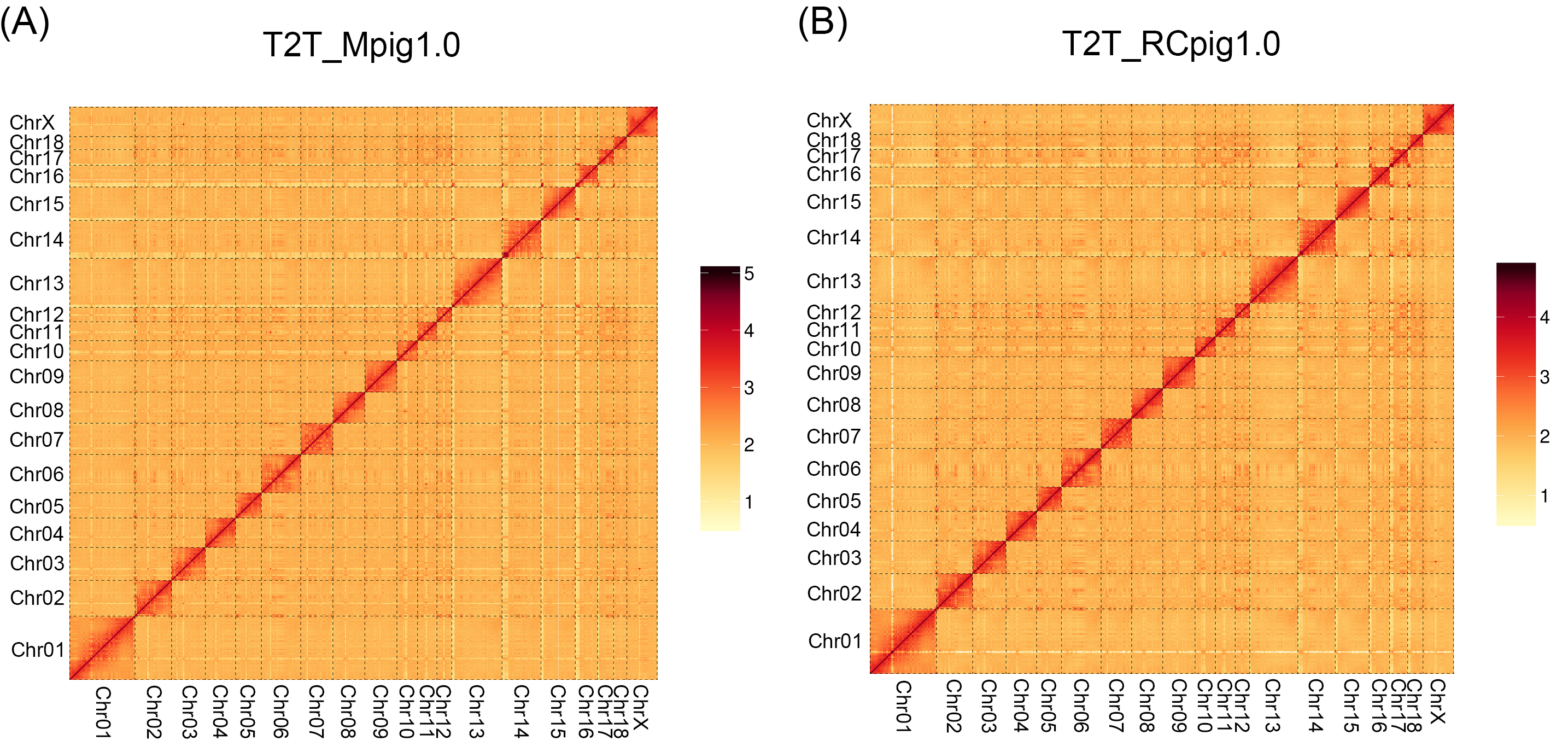


**Figure S2. Heatmaps of chromosomal interactions of T2T_Mpig1.0 and T2T_RCpig1.0 genomes.**


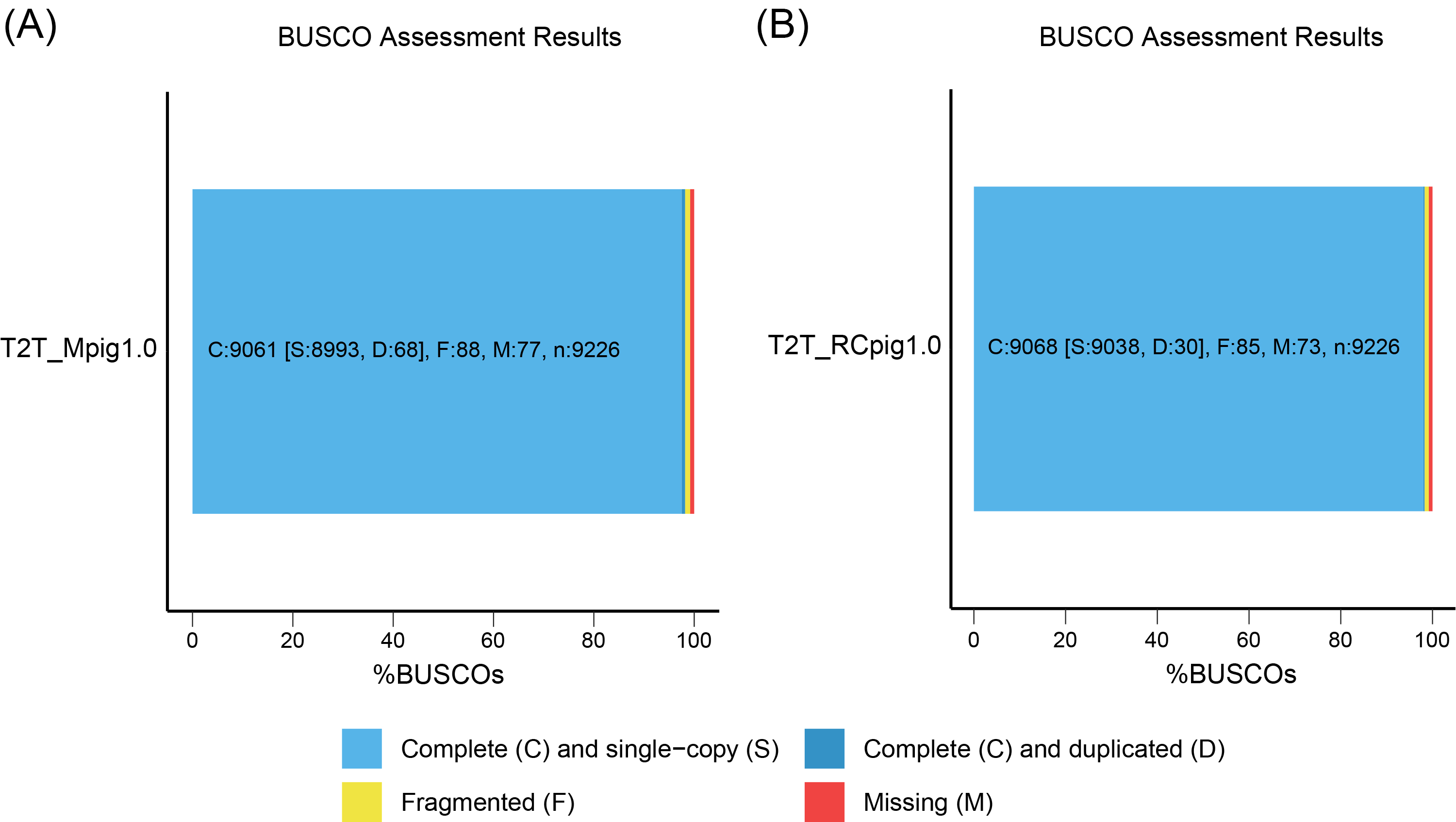


**Figure S3. Assessment of the genomic completeness of T2T_Mpig1.0 and T2T_RCpig1.0 using the BUSCO tool.** The completeness scores were calculated using the mammalia_odb10 database.


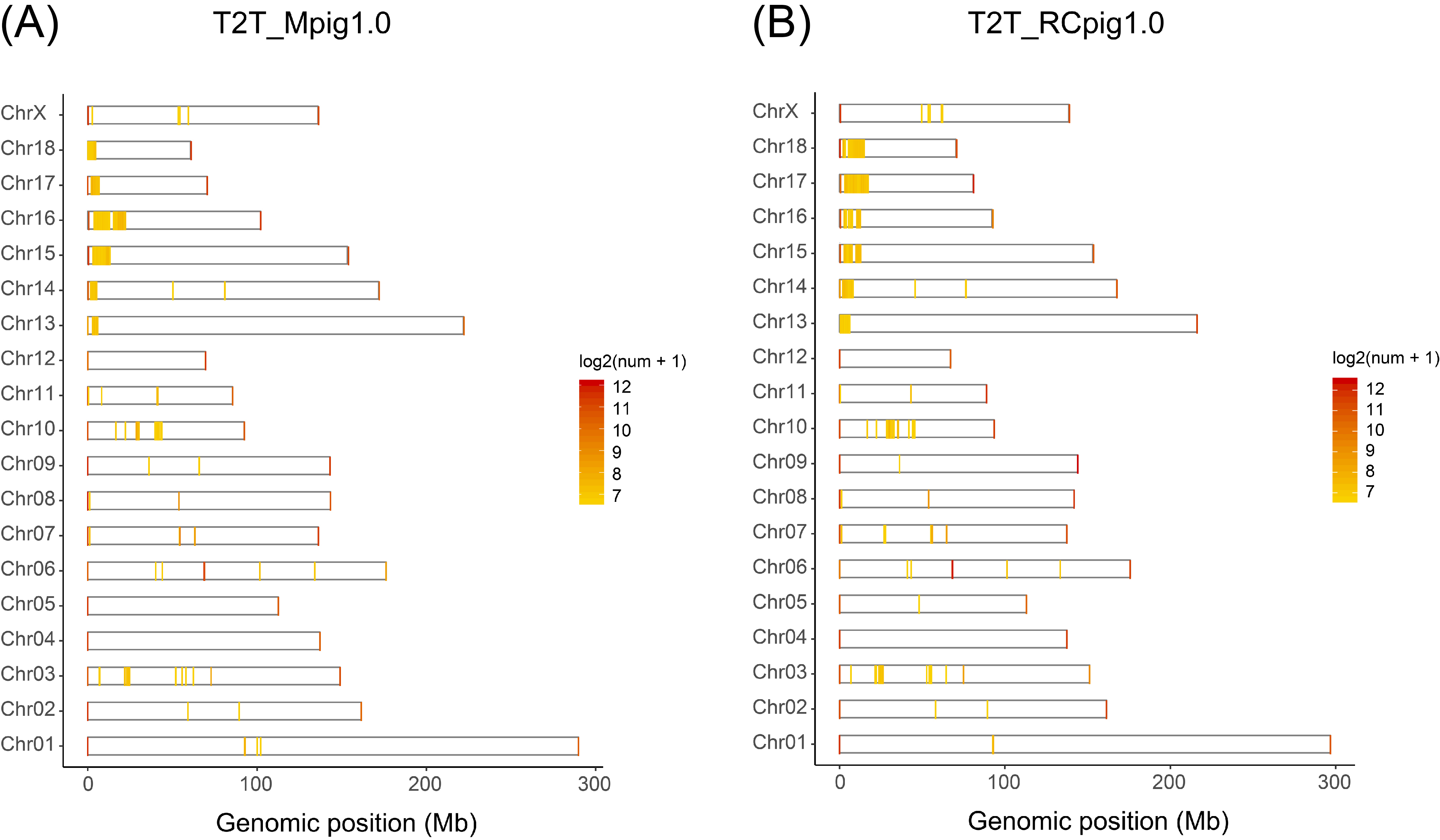


**Figure S4.** **Heatmaps showing the distribution of telomeres in T2T_Mpig1.0 and T2T_RCpig1.0 genomes.** Telomere sequences were identified within a 100-kb window using Tidk software.


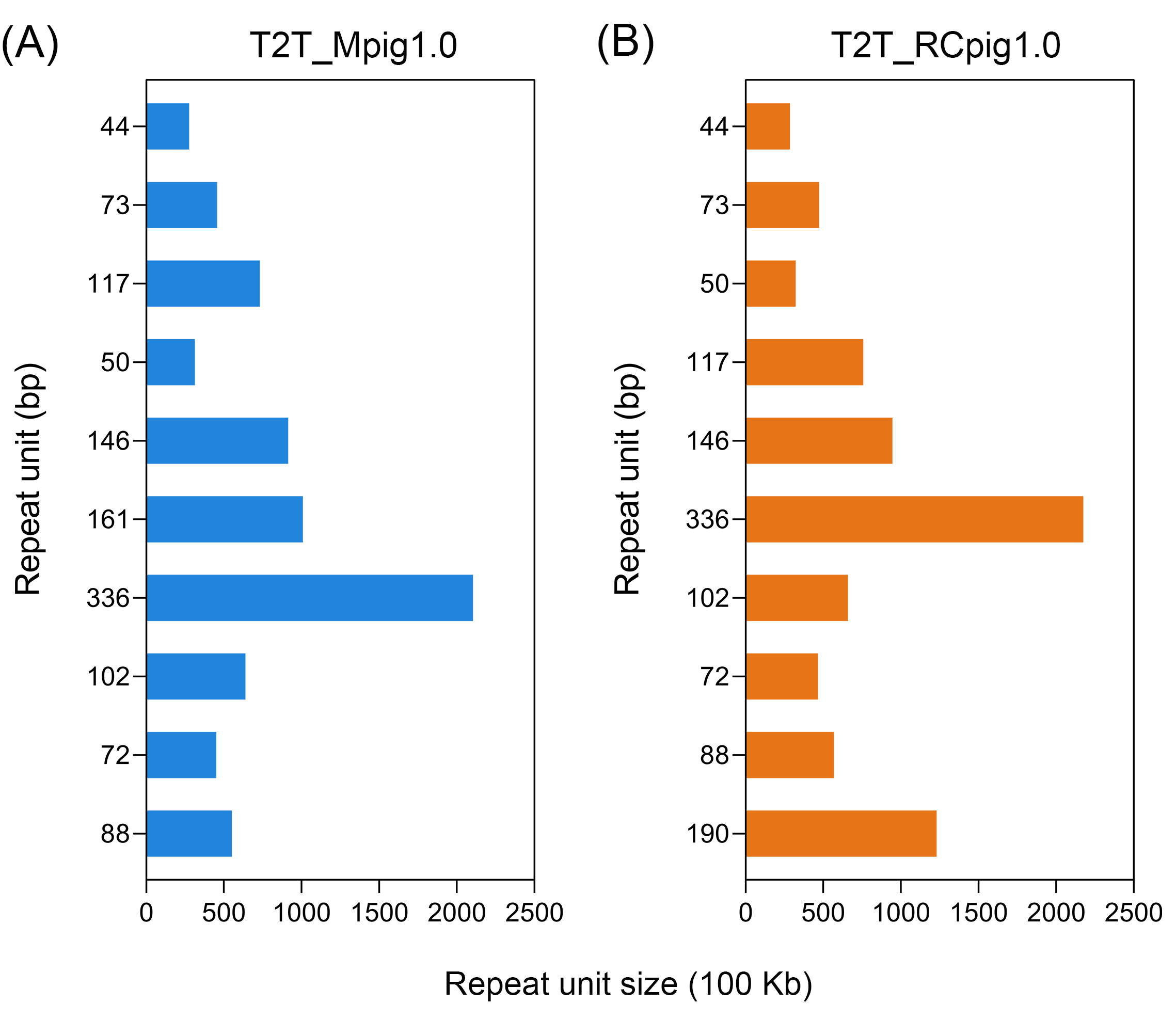


**Figure S5. Total length of the Top ten repeat units in the genomes.**

**
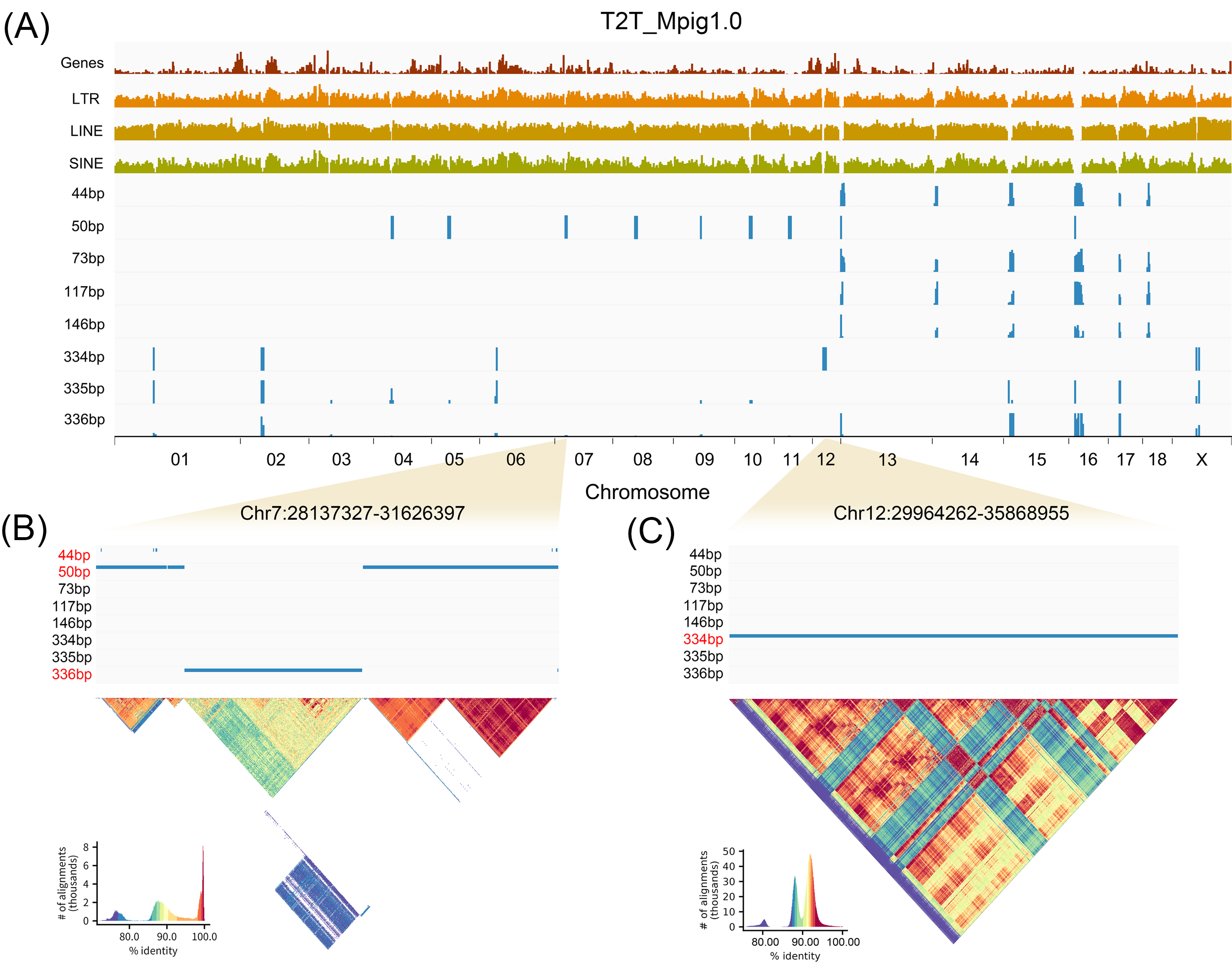
**

**Figure S6. Characterization of repeating units in the centromere region of T2T_Mpig1.0.** (A) Distribution of coding genes, TEs (LTRs, LINEs, and SINEs), and different repeat units in T2T_Mpig1.0. (B-C) Visualization of the predicted centromere regions of (B) chromosome 7 and (C) chromosome 12. The heatmaps show the pairwise sequence identity (%) between 4-kb sequences. The presence of repetitive units in the centromeres is marked in red.

**
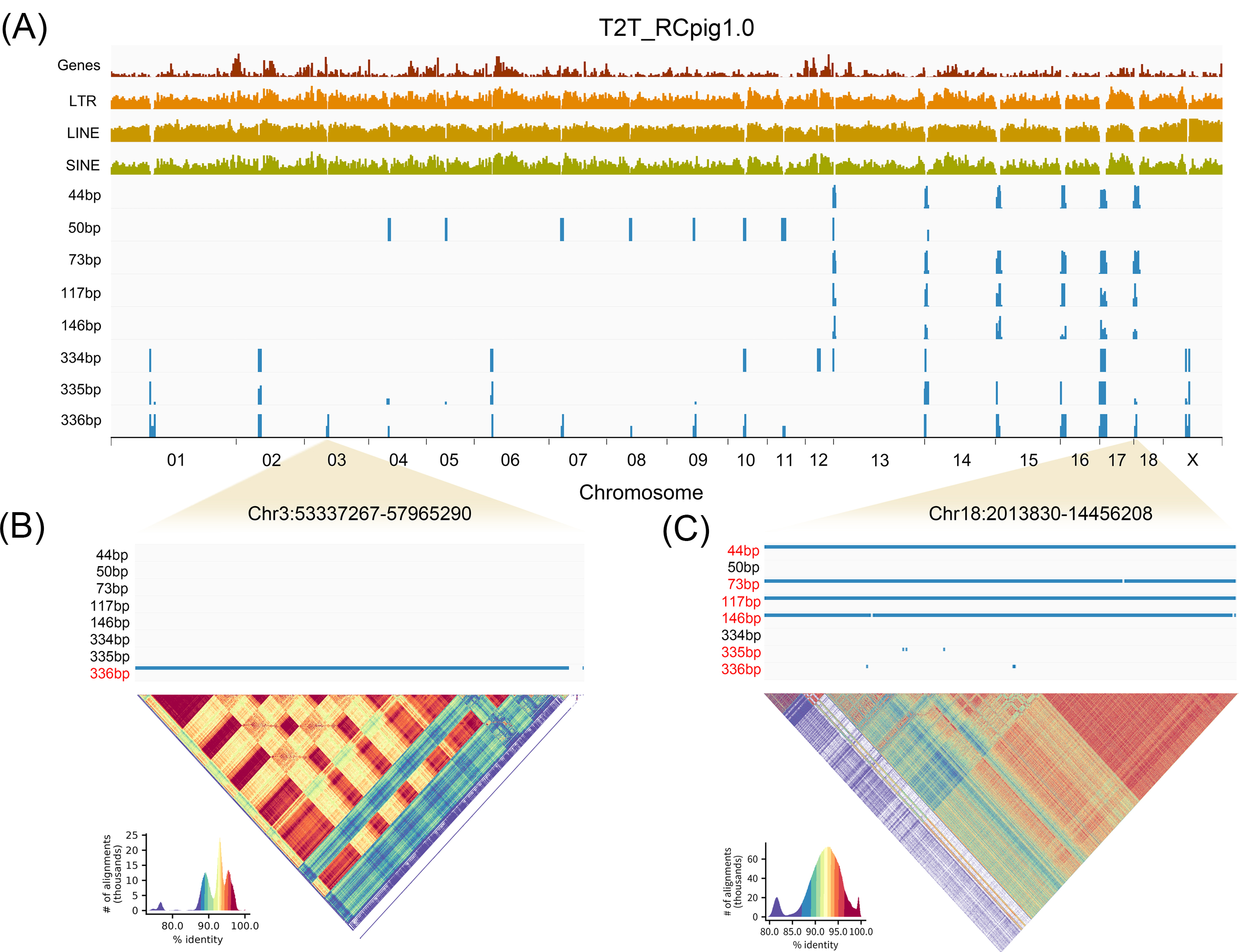
**

**Figure S7. Characterization of repeating units in the centromere region of T2T_RCpig1.0.** (A) Distribution of coding genes, TEs (LTRs, LINEs, and SINEs), and different repeat units in T2T_RCpig1.0. Visualization of the predicted centromere regions of (B) chromosome 3 and (C) chromosome 18. The heatmaps show the pairwise sequence identity (%) between 4-kb sequences. The presence of repetitive units in the centromeres is marked in red.

**Figure S8. Presence and absence information of pan-gene families in the 14 pig genomes**.


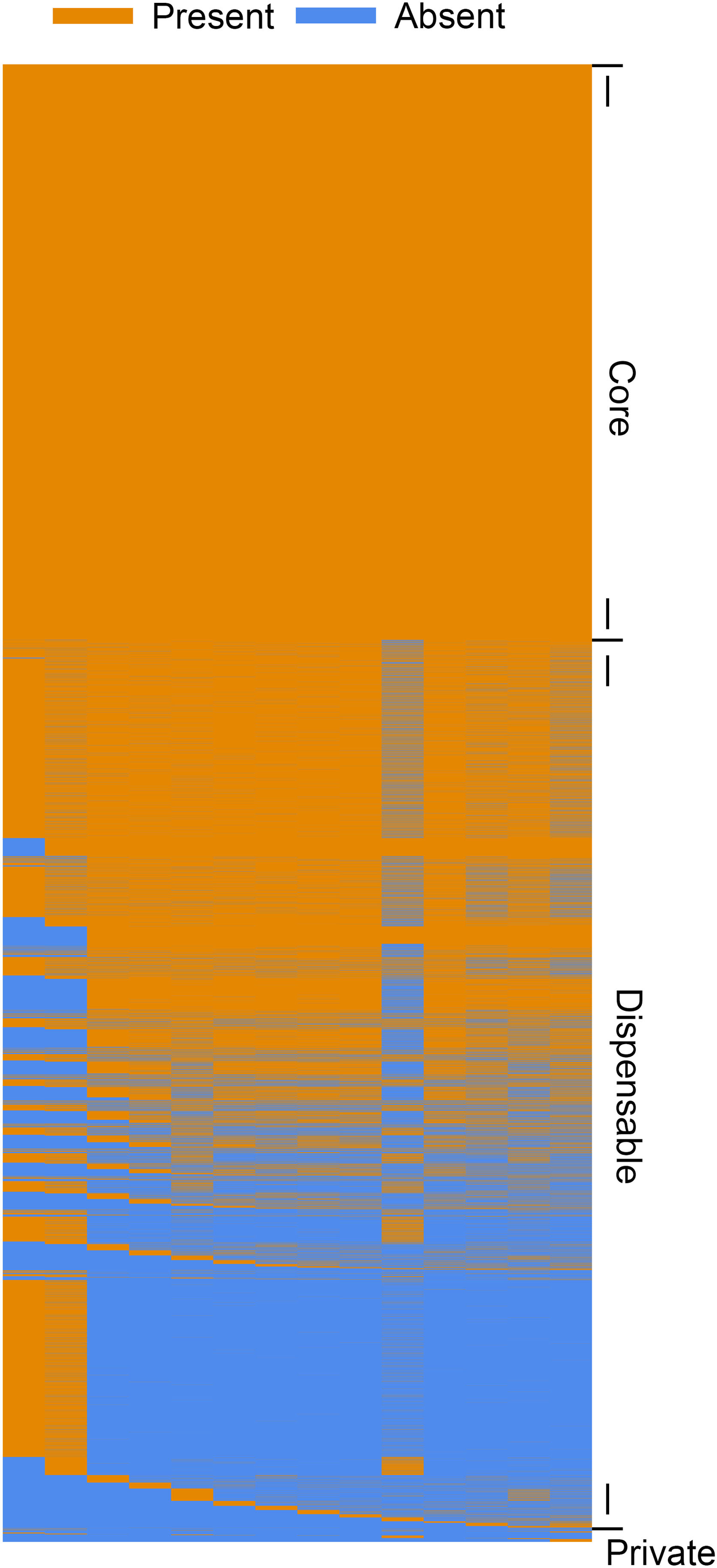

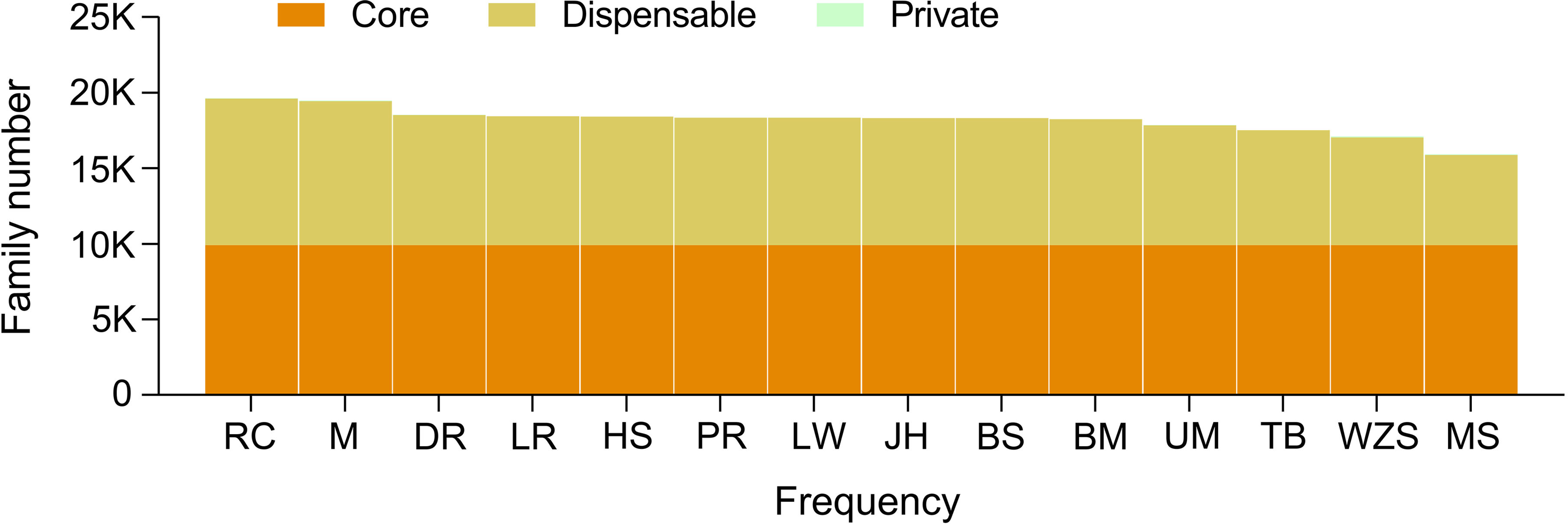


**Figure S9.** **Number of gene families in each category across individual genomes.**


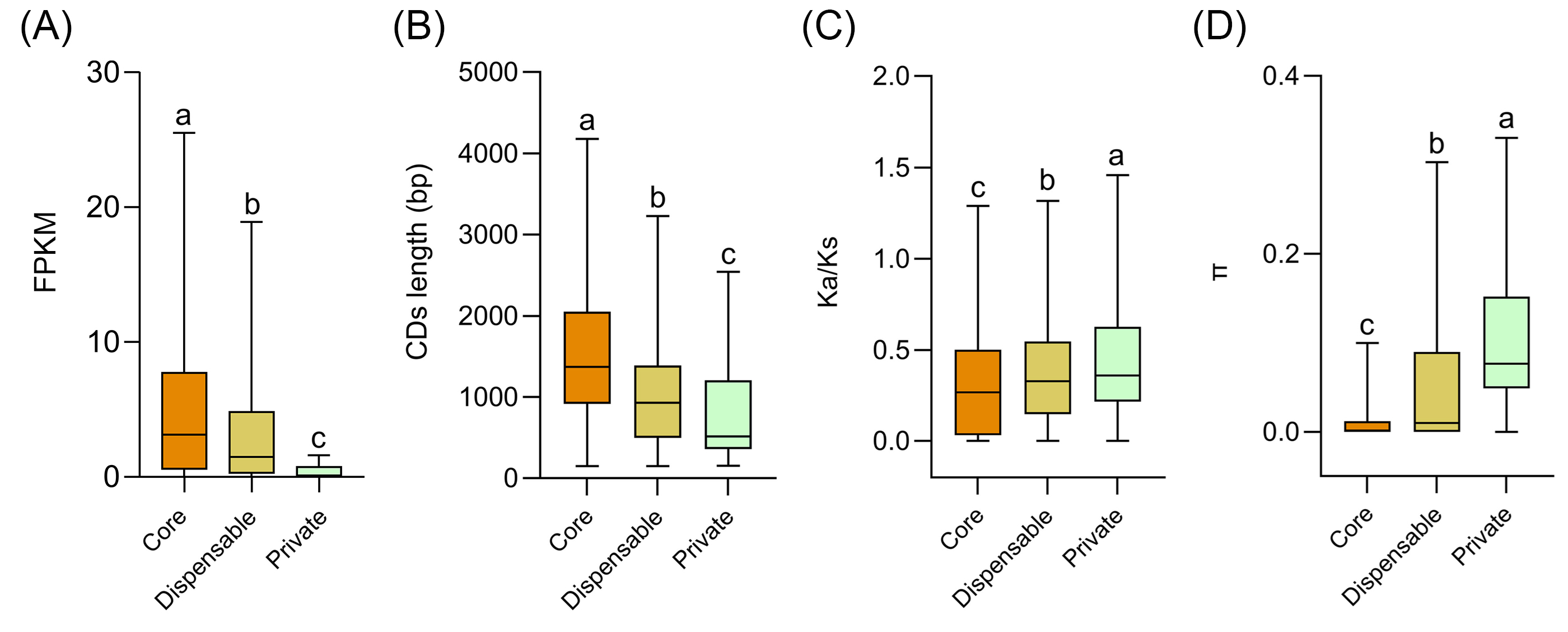


**Figure S10. Comparative analysis of (A) FPKM, (B) CDS length, (C) Ka/Ks ratios, and (D) π values among core, dispensable, and private genes.** The boxplots display interquartile ranges (IQR), with whiskers extending up to 1.5 × IQR. Centerlines represent the medians. Statistical comparisons were conducted using the Student-Newman-Keuls test with α = 0.001.


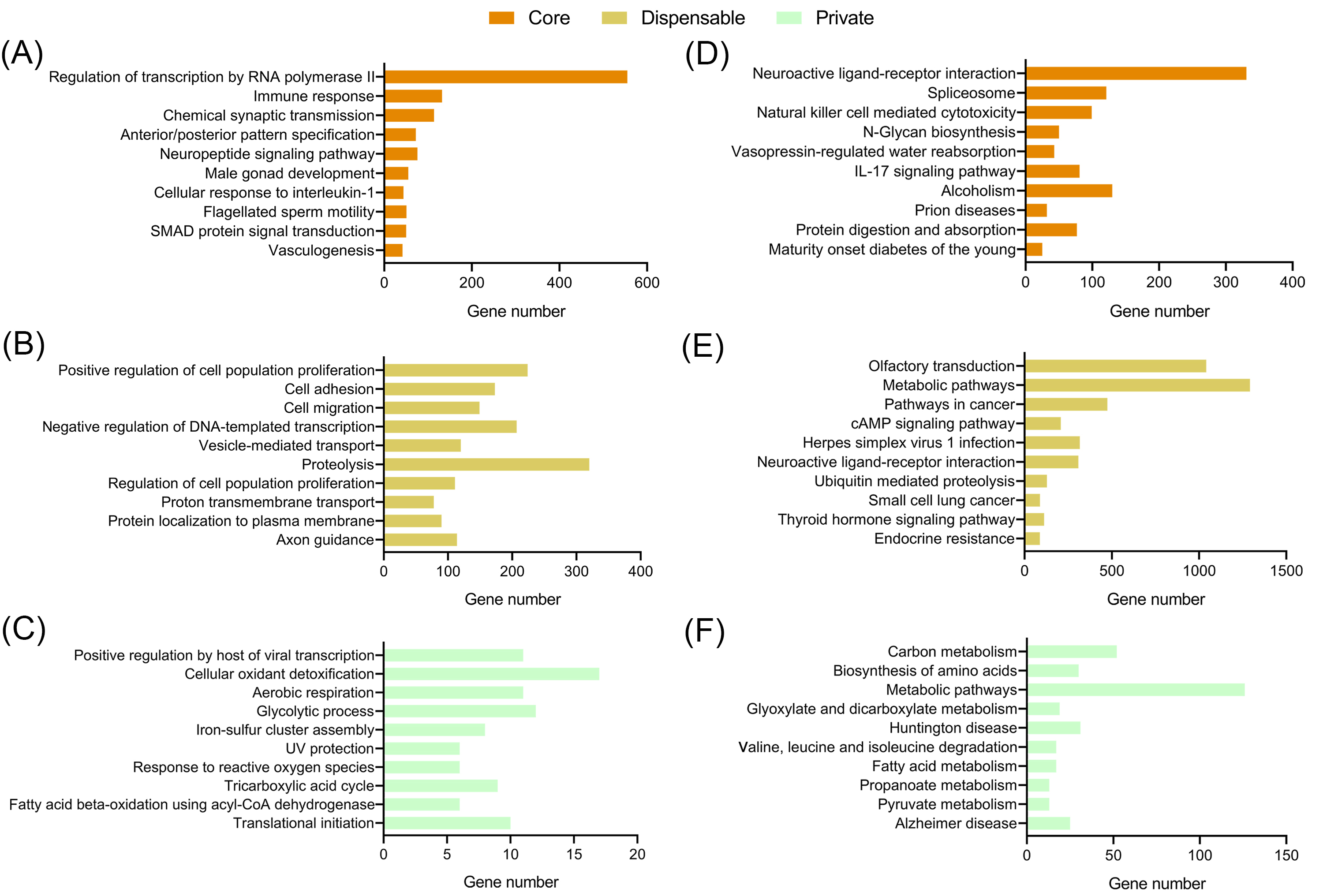


**Figure S11. GO and KEGG enrichment analyses for core, dispensable, and private genes.** (A-C) The Top 10 GO terms for core, dispensable, and private genes. (D-F) The Top 10 KEGG pathways for core, dispensable, and private genes. All terms and pathways are ordered from top to bottom based on the *p*-value.

**Figure S12. Geographic distribution of the pig breeds used in this study.**


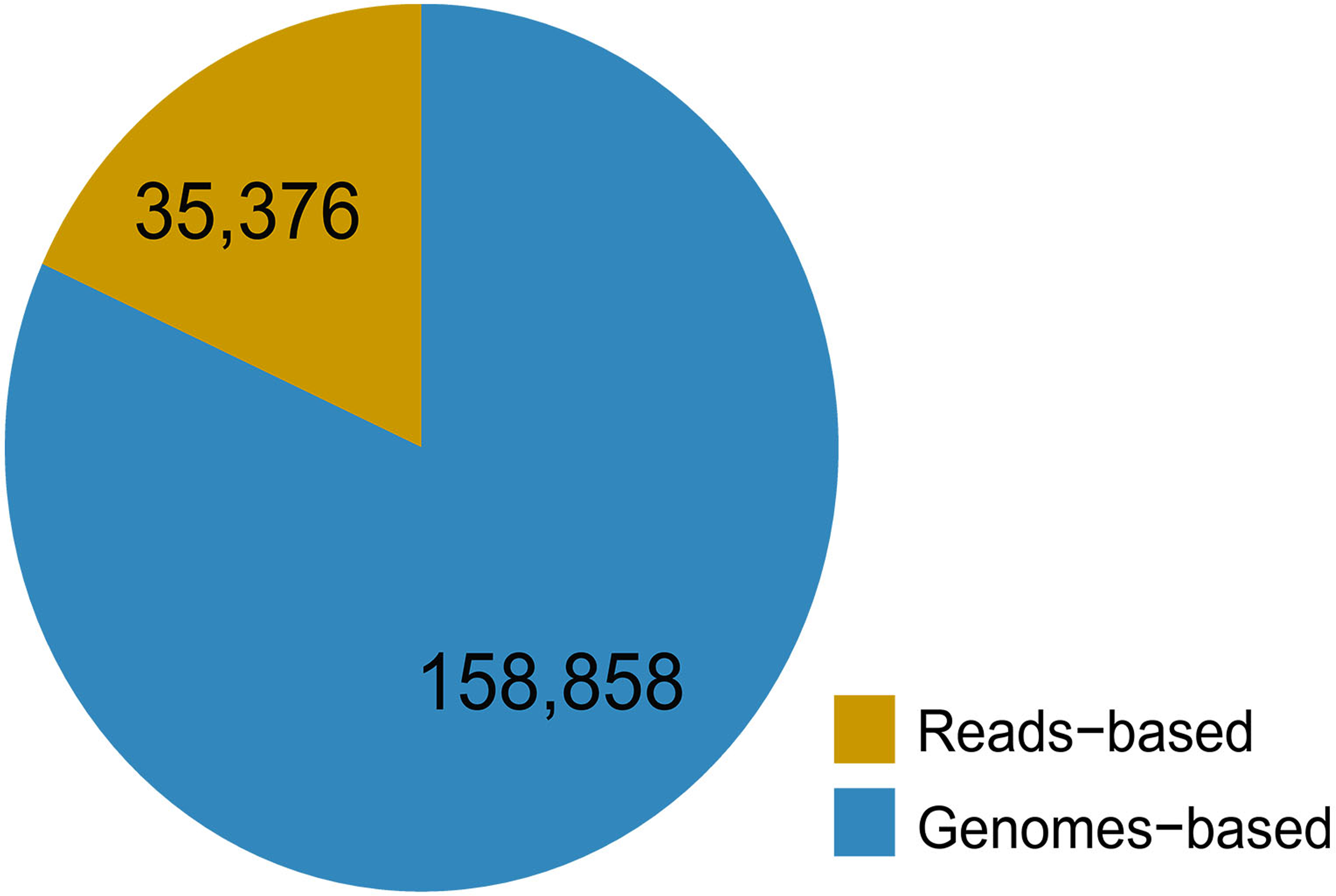


**Figure S13.** **Pie chart showing the proportion of SVs identified through genome alignment and read mapping strategies.** Arabic numerals represent the total number of SVs.

**
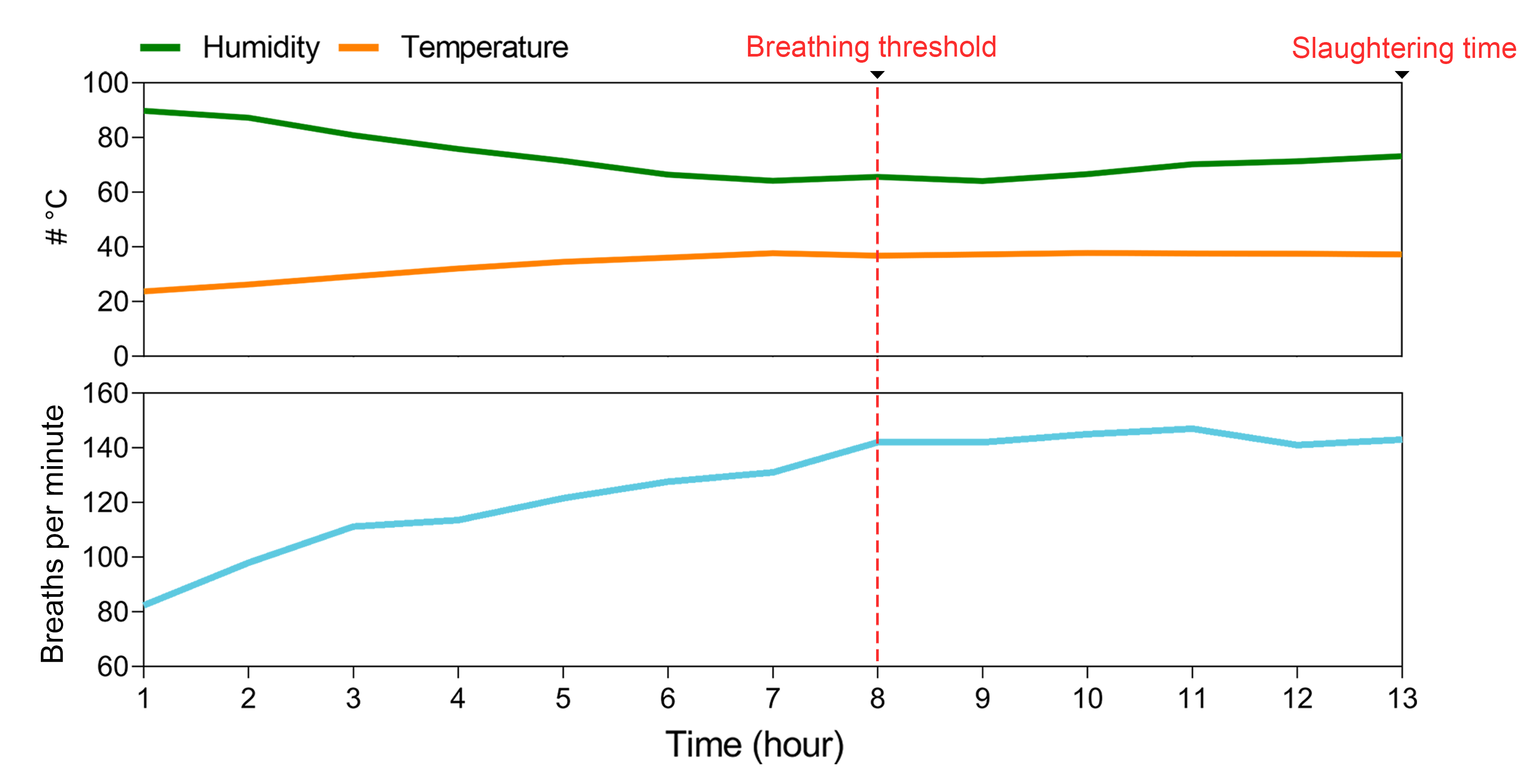
**

**Figure S14. Hourly measurements of temperature, humidity, and respiratory rate during heat stress in Rongchang pigs.** Slaughter time was determined when the breathing rate stabilized after 5 hours.


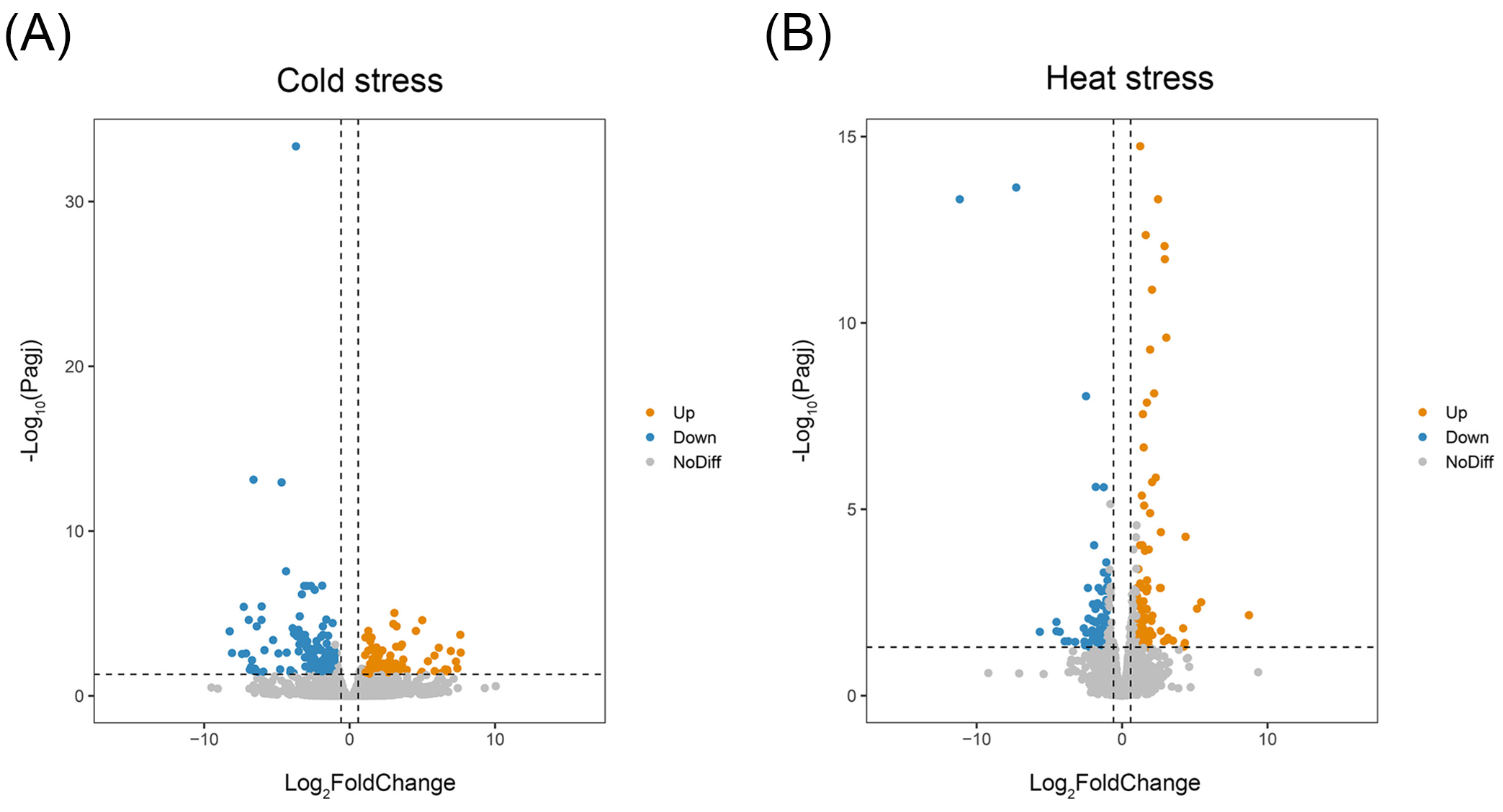


**Figure S15. Volcano plots of differentially expressed genes (DEGs) under cold stress for Min pigs and heat stress for Rongchang pigs.**


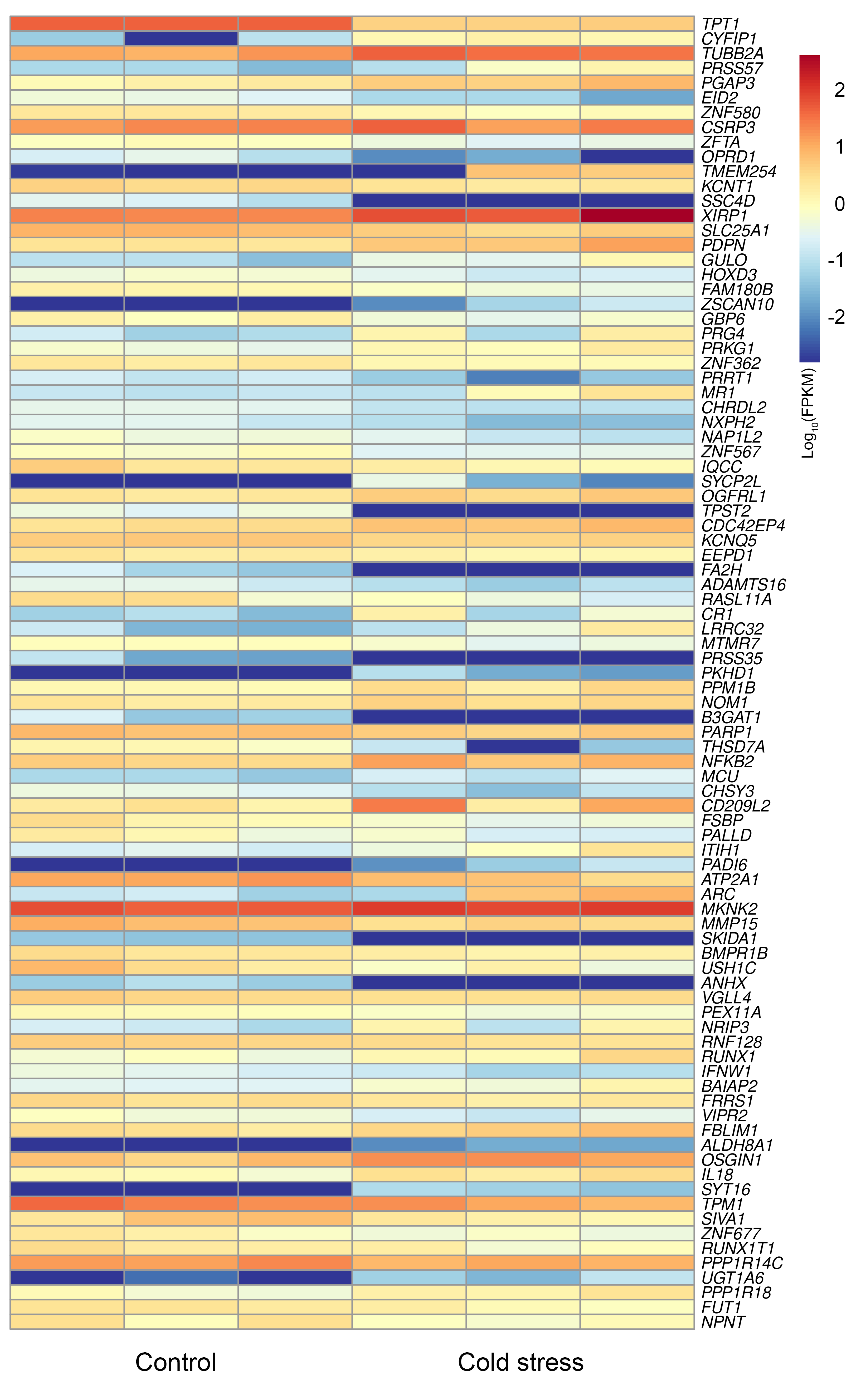


**Figure S16. The missing 89 DEGs reported in a previous study.** Gene expression levels were normalized using FPKM and subsequently log-transformed to the log_10_ scale.

**
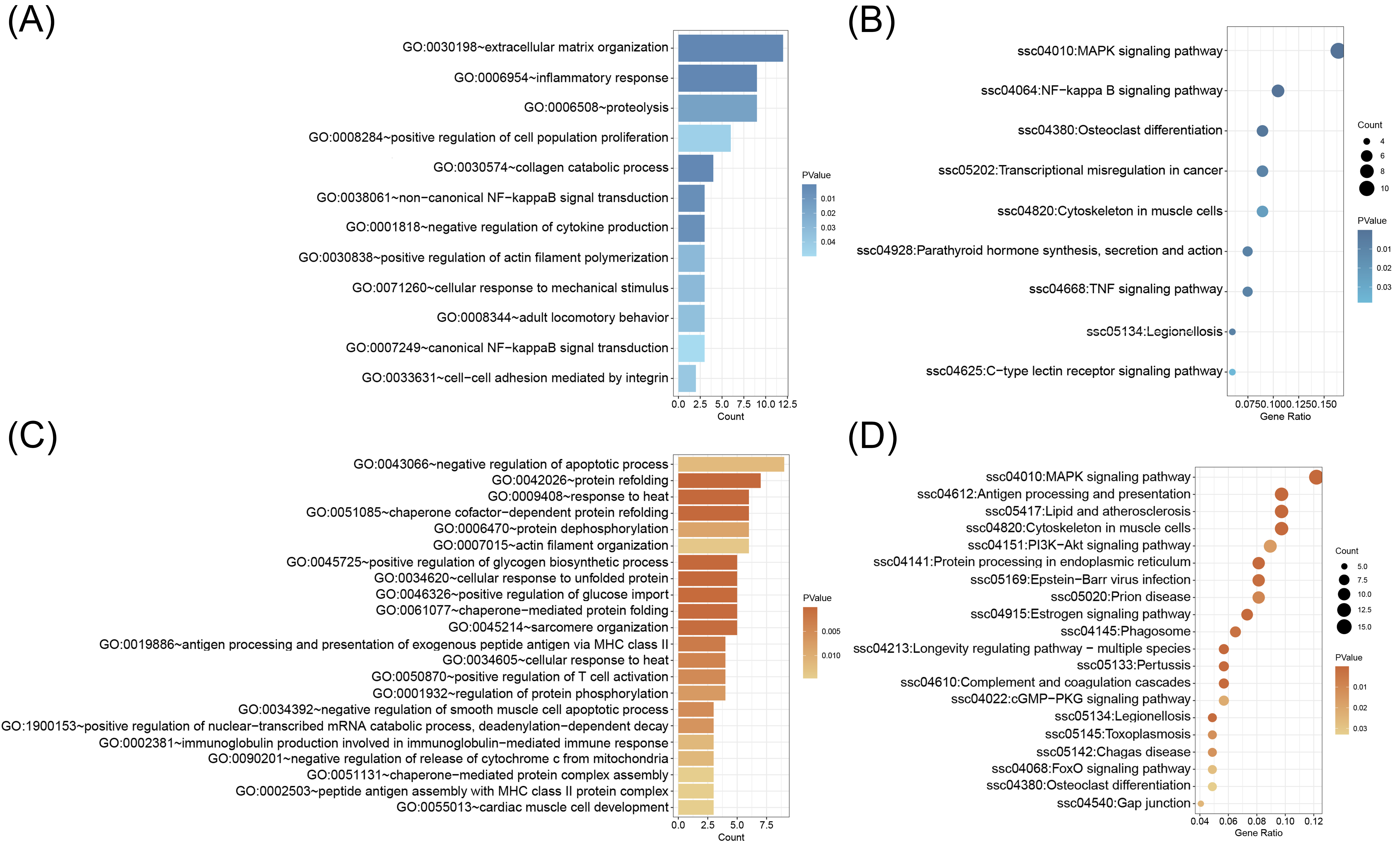
**

**Figure S17. DEGs involved in GO terms and KEGG pathways under cold and heat exposures. (**A-B) GO and KEGG enrichment analysis of cold exposure. (C-D) GO and KEGG enrichment analysis of hot exposure. GO terms and KEGG pathways with *p* values < 0.05 were retained.


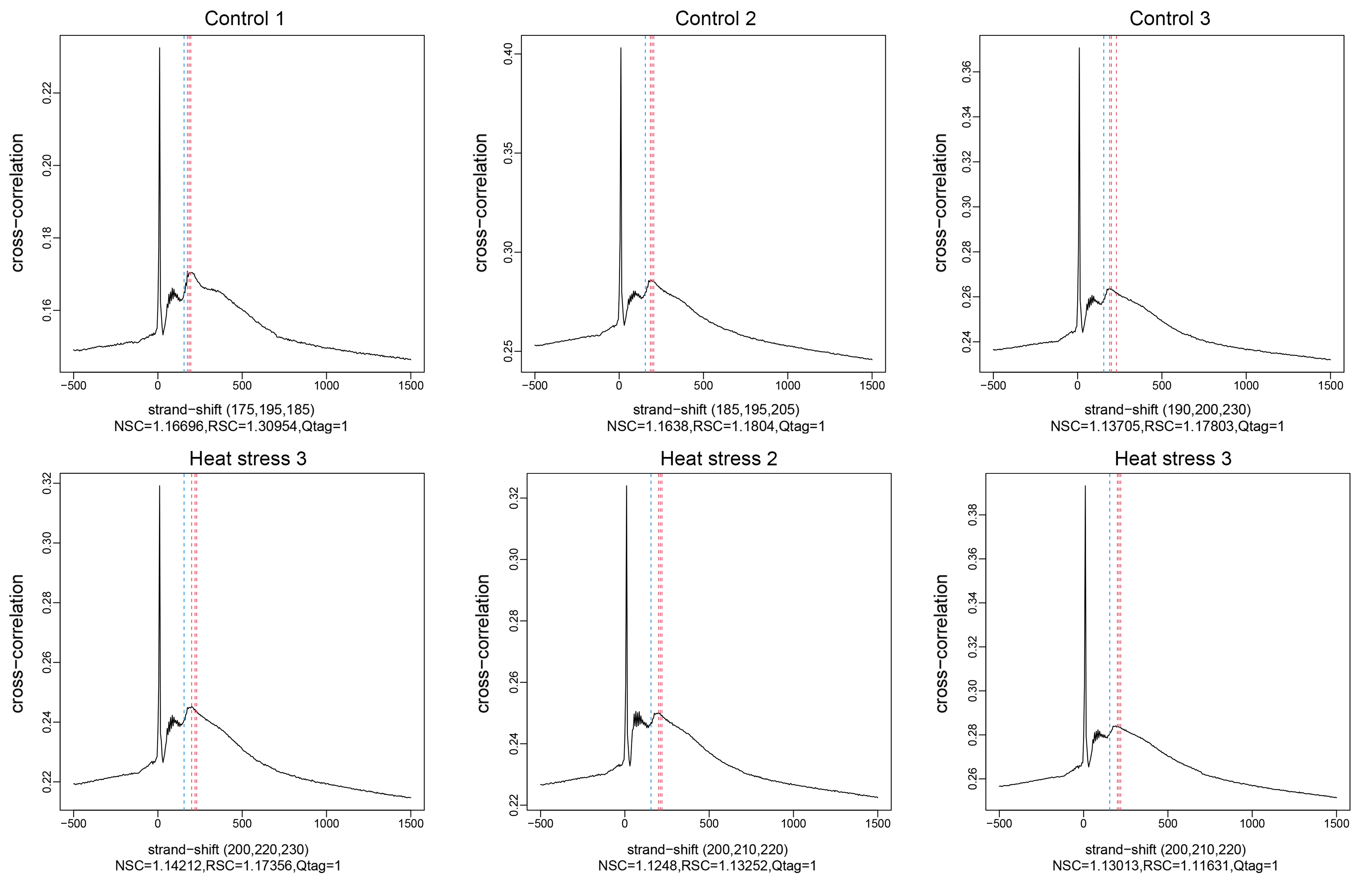


**Figure S18. Assessment of cross-correlation values (NSC and RSC) in heat stress and control samples.**


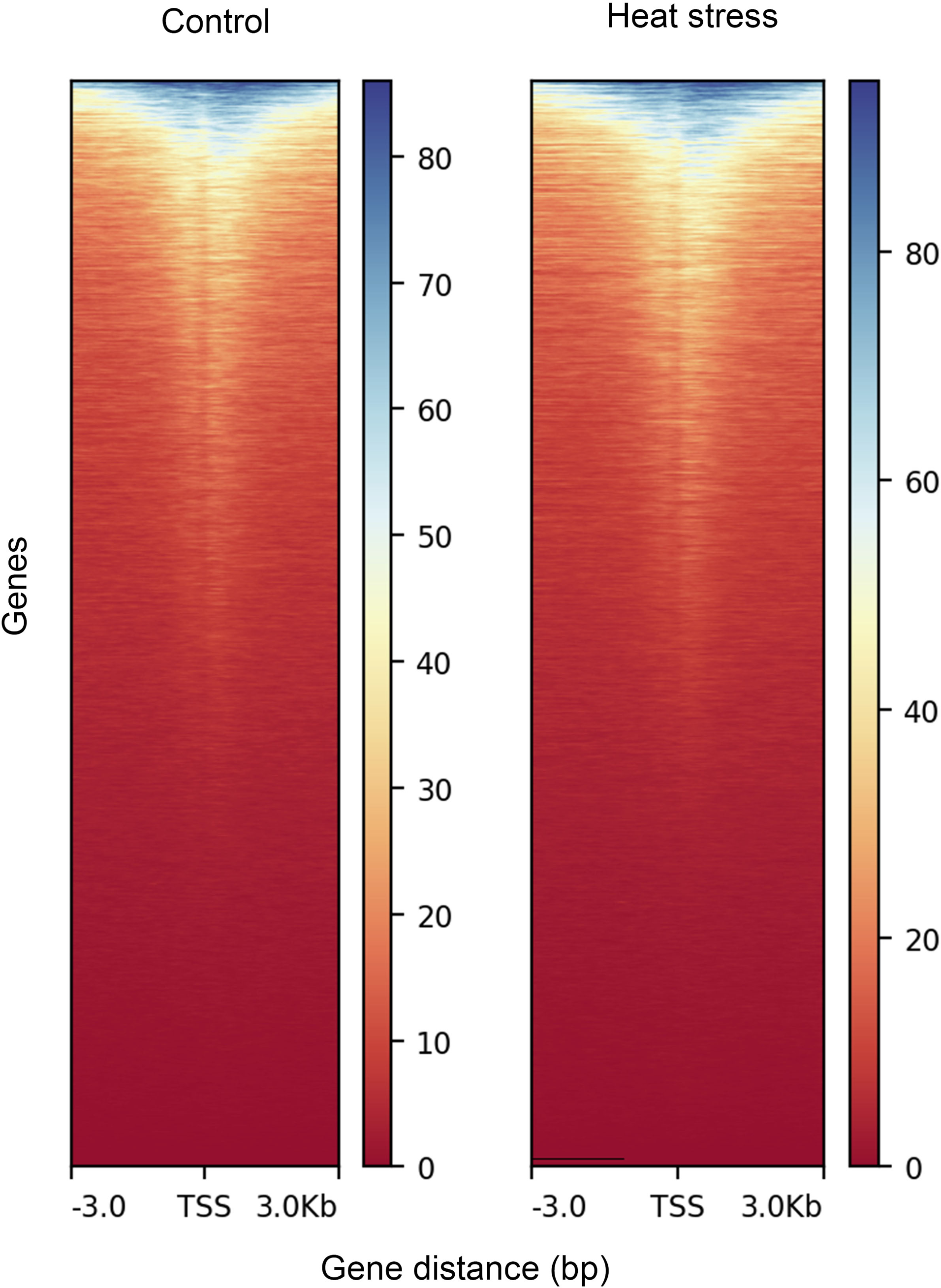


**Figure S19. Functional enrichment analysis of genes related to H3K27ac modification in skeletal muscle of Rongchang pigs from control and heat stress groups.**

**
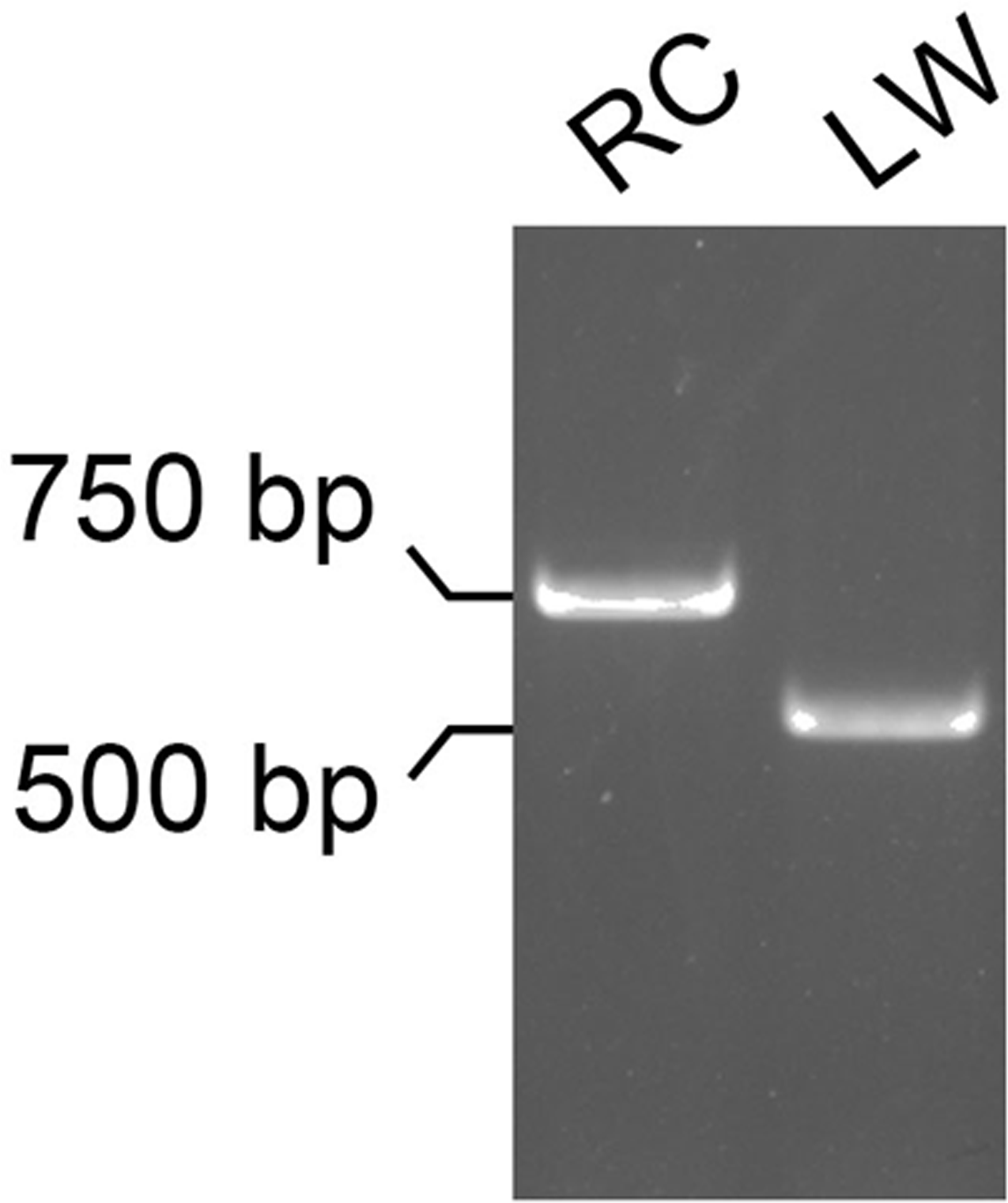
**

**Figure S20.** **Validation of SV upstream of *GIMAP6* by electrophoresis.** A Rongchang pig (RC) and a Large White pig (LW) were selected for gel electrophoresis analysis.

**
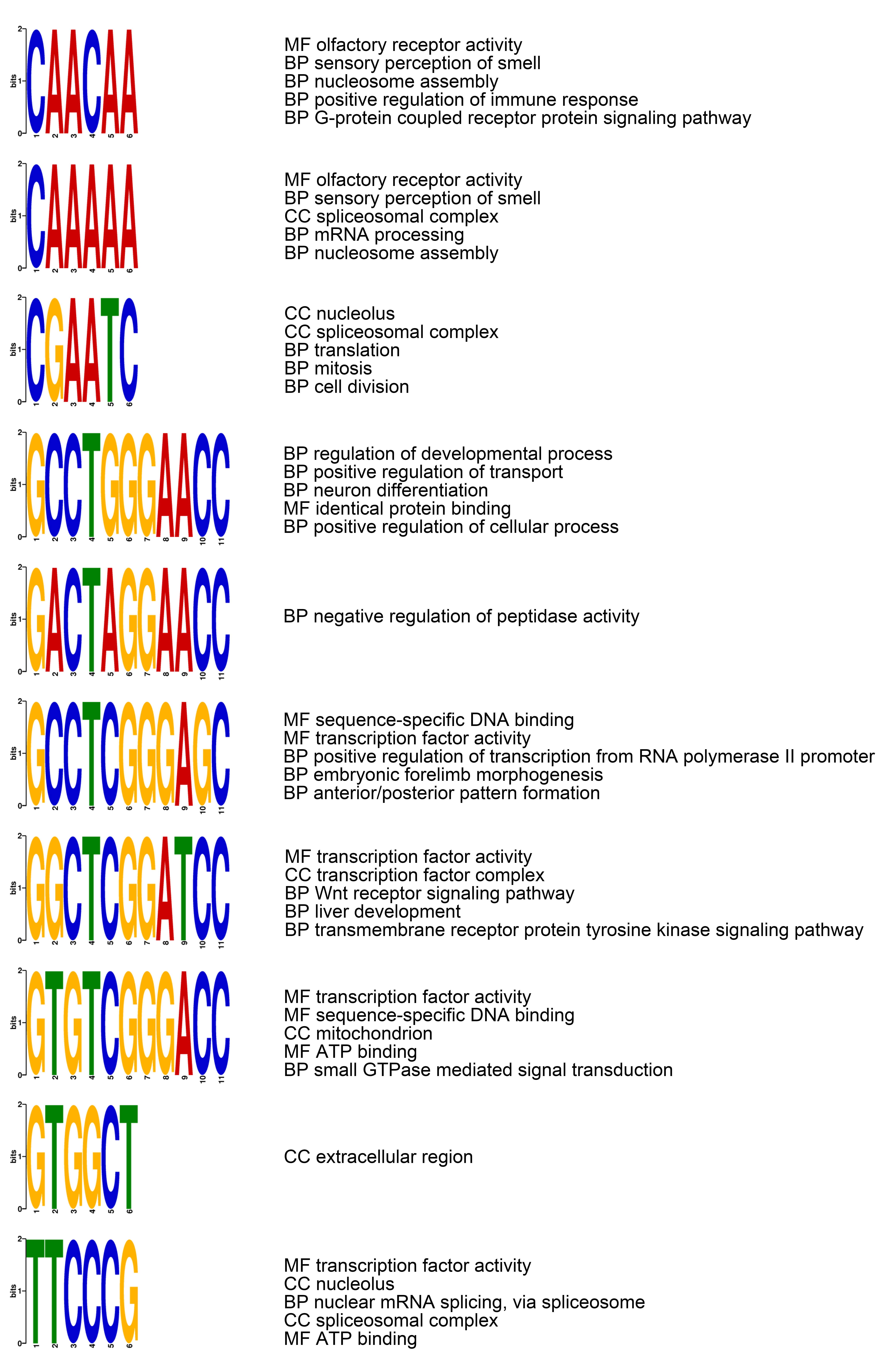
**

**Figure S21. Prediction of motifs in the DEL of the promoter region of *GIMAP6* and Top five GO terms.**

**
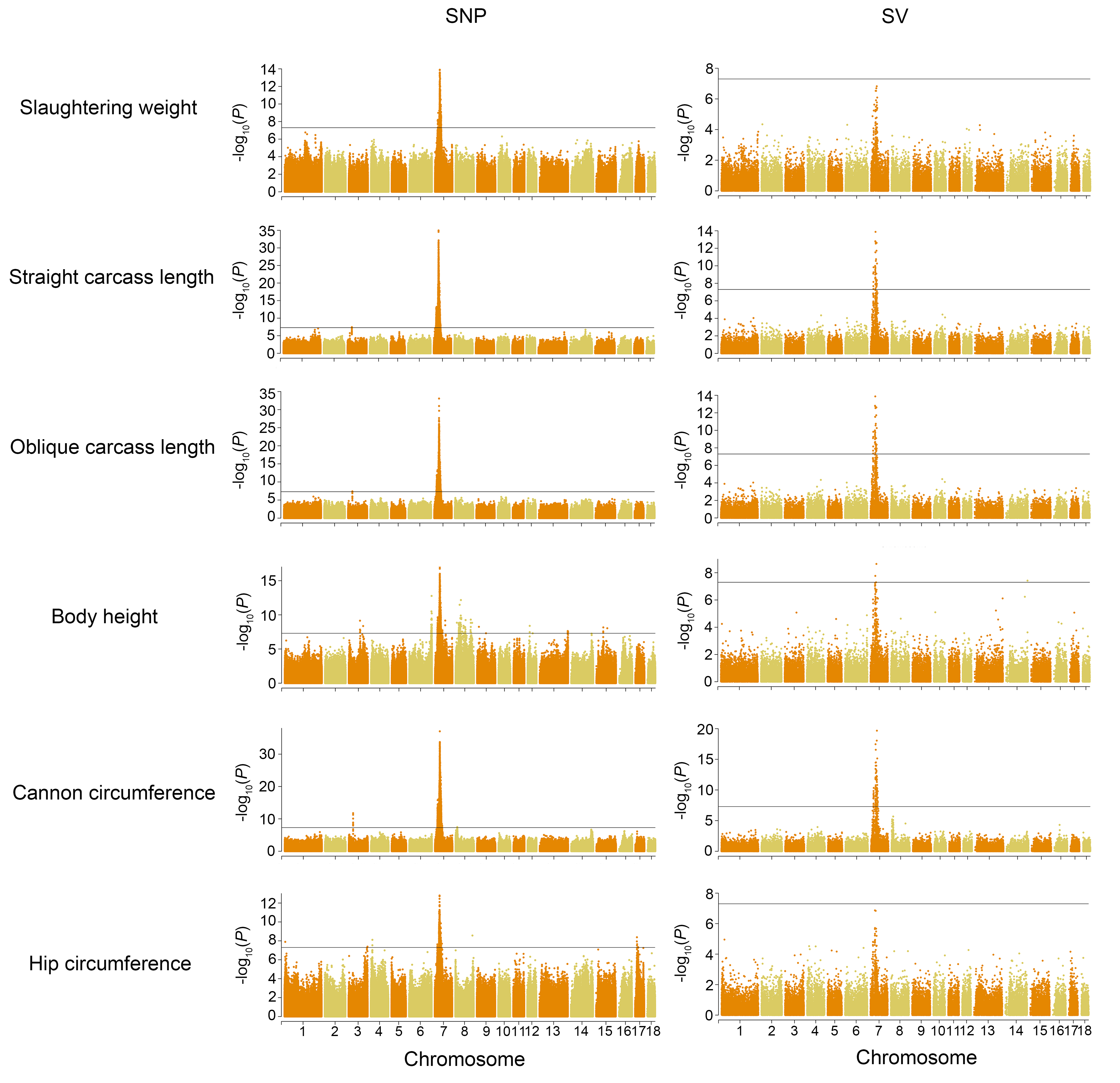
**

**Figure S22. Manhattan plots showing GWAS for six carcass traits based on SNPs and SVs.** The significance threshold line used was 5 × 10^-8^.
